# Antibody-protein binding and conformational changes: identifying allosteric signalling pathways to engineer a better effector response

**DOI:** 10.1038/s41598-020-70680-0

**Published:** 2020-08-13

**Authors:** Mohammed M. Al Qaraghuli, Karina Kubiak-Ossowska, Valerie A. Ferro, Paul A. Mulheran

**Affiliations:** 1 Department of Chemical and Process Engineering, University of Strathclyde, Glasgow, G1 1XJ UK; 2 SiMologics Ltd. The Enterprise Hub, Level 6 Graham Hills Building, 50 Richmond Street, Glasgow, G1 1XP UK; 3 Department of Physics, University of Strathclyde, Glasgow, G4 0NG UK; 4 Strathclyde Institute of Pharmacy and Biomedical Sciences, University of Strathclyde, 161 Cathedral Street, Glasgow, G4 0RE UK

**Keywords:** Proteins, Biological models, Biologics, Antibody fragment therapy, Antibody therapy, Computational models, Protein analysis, Protein function predictions, Protein structure predictions

## Abstract

Numerous monoclonal antibodies have been developed successfully for the treatment of various diseases. Nevertheless, the development of biotherapeutic antibodies is complex, expensive, and time-consuming, and to facilitate this process, careful structural analysis beyond the antibody binding site is required to develop a more efficacious antibody. In this work, we focused on protein antigens, since they induce the largest antibody changes, and provide interesting cases to compare and contrast. The structures of 15 anti-protein antibodies were analysed to compare the antigen-bound/unbound forms. Surprisingly, three different classes of binding-induced changes were identified. In class (B1), the antigen binding fragment distorted significantly, and we found changes in the loop region of the heavy chain’s constant domain; this corresponds well with expected allosteric movements. In class (B2), we found changes in the same loop region without the overall distortion. In class (B3), these changes did not present, and only local changes at the complementarity determining regions were found. Consequently, structural analysis of antibodies is crucial for therapeutic development. Careful evaluation of allosteric movements must be undertaken to develop better effector responses, especially during the transformation of these antibodies from small fragments at the discovery stage to full antibodies at the subsequent development stages.

## Introduction

Diversity in the vertebrate immune system is a critical factor in the continuous protection against a plethora of invading pathogens. In this context, antibodies, which are produced by B-lymphocytes, play a pivotal role to neutralise these invading agents and eliminate them through different immunological mechanisms. This is evident in their ability to recognise various molecular signatures, ranging from proteins, peptides, nucleic acids, lipids, or small haptens with a molecular weight < 1,000 Da^1^.

An antibody is composed of two Fragment antigen binding (Fab) regions and one Fragment crystallisable (Fc) region^2,3^. The Fab fragment is responsible for antigen recognition through its variable sites, while the Fc region can propagate a series of effector responses^4^. The antigen recognition process is accomplished by the complementarity determining regions (CDRs) located at the tips of the Fabs, occasionally being supported by a few residues from the framework regions (highly conserved regions that are responsible for acting as a scaffold for the CDRs). In general, an antibody should discriminate between self-molecules (produced by an organism) and exogenous foreign antigens (like viruses and bacteria) to restrict its function against the external antigens, and demonstrate the required tolerance to self-antigens. The inability to distinguish between these interaction processes might explain several autoimmune diseases.

The six CDRs comprise ~ 70 residues out of the ~ 230 amino acids in each chain of the Fab fragment^5^, and form a continuous surface of approximately 2,800 Å^2^^6^. Webster et al. initially correlated the binding sites surface topography to various antigen types^7^. These shapes include pockets to accommodate small haptens, grooves to enclose peptides, and flat surfaces to grasp large protein surfaces^8^. These surface arrangements were confirmed by measurements of the solvent accessible surface area, which were estimated to be 200–400 Å^2^, 400–600 Å^2^ and ~ 800 Å^2^ for anti-haptens, anti-peptides, and anti-protein antibodies, respectively^9^.

It is widely proposed that an antibody-antigen interaction can use either specific or nonspecific interfaces where the surfaces may mould to fit each other, following a “lock-and-key” or “hand-shake” (induced fit) format, respectively. The generated antibodies’ affinity is largely dependent on the number and type of amino acids that form the CDRs loops, and determine the binding site surface topography^10^. The antigen-binding region (paratope) and the antigenic determinant (epitope) are held together by non-covalent forces, which also dictate the affinity of these antibodies^11^. This interaction is also orchestrated by complementarity in the paratope-epitope charge and shape^1^. Somatic gene-recombination and mutations, antibody class-switching, and heavy and light chain dimerisation, are mechanisms that can craft the malleable binding sites to accommodate a wide range of antigens^12–15^.

Despite the significant interest of the biopharmaceutical industry in generating and crafting antibody binding sites with the highest affinity, it is equally crucial to understand the conformational changes beyond these binding sites. This will ensure successful and compatible transformation of small antibody fragments, such as single-chain variable fragment (scFv), single chain antibody fragments (scAbs), and Fabs, at the early developmental stages to full antibodies, without losing their high affinities or their effector functions. Consequently, understanding how the interaction of an antibody with its antigen propagates a signal to the Fc region (the allosteric model) is of critical importance to the engineering of antibodies and the development of new therapeutics.

In order to address the above question, we have analysed various Fab crystal structures of IgG antibodies, crystallised in free and antigen-complexed formats, that are available in the Protein Data Bank (PDB) (Table 1). Sela-Culang et al.^16^ reported that antibody binding to proteins induces larger conformational changes than binding to smaller peptides, associating this with an allosteric signalling response. Focusing on protein antigens as a potential target-class for several biotherapeutic programmes^17^, and by detailed inspection of available structures that are amenable to analysis, we have found that the allosteric mechanism proposed in^16^ is not universally observed. While we could identify cases where the antigen binding induces a clear allosteric signalling pathway, we also found cases when the binding results only in local changes. The analysis will help clarify the type of structural change required for the antibody to achieve its desired properties when under development as a new therapeutic.

**Table 1:** PDB couples examined in this work.

PDB couples	Species	Target	Development
3G6A/3G6D	Homo sapiens	Interleukin 13 (IL-13)	CNTO 607 (monoclonal antibody originally isolated from the MorphoSys phage display library HuCAL)
3HMW/3HMX	Homo sapiens	Interleukin 12 (IL-12)	Ustekinumab or CNTO 1,275 (developed by Centocor, from transgenic mice using genetic engineering techniques)
3EO9/3EOA	Homo sapiens	Lymphocyte function-associated antigen 1 (LFA-1)I domain, Form I	Efalizumab (developed by Genentech/Xoma, as a humanized antibody produced in a Chinese hamster ovary mammalian cell expression system)
3EO9/3EOB	Homo sapiens	Lymphocyte function-associated antigen 1 (LFA-1) I domain, Form II	As above
1MLB/1MLC	Mus musculus	Chicken egg-white lysozyme	D44.1 (Mouse monoclonal antibody)
1DQQ/1DQJ	Mus musculus	Hen egg white lysozyme	HYHEL-63 (Mouse monoclonal antibody)
5BVJ/5BVP	Homo sapiens	Interleukin 1 beta (IL-1β)	Canakinumab or ACZ885 (fully human antibody developed by Novartis, through UltiMab technology (Medarex) using transgenic mice)
2FJF/2FJG	Homo sapiens	Vascular endothelial growth factor (VEGF)	Synthetic antibody phage libraries

## Results

Due to the large amount of data, only the most characteristic observations and conclusions are included in the manuscript while the entire data set supporting these, as well as further details of the analyses, can be found in Supplementary Materials.

### Antibody domain orientation changes on antigen binding

**Scheme 1:**
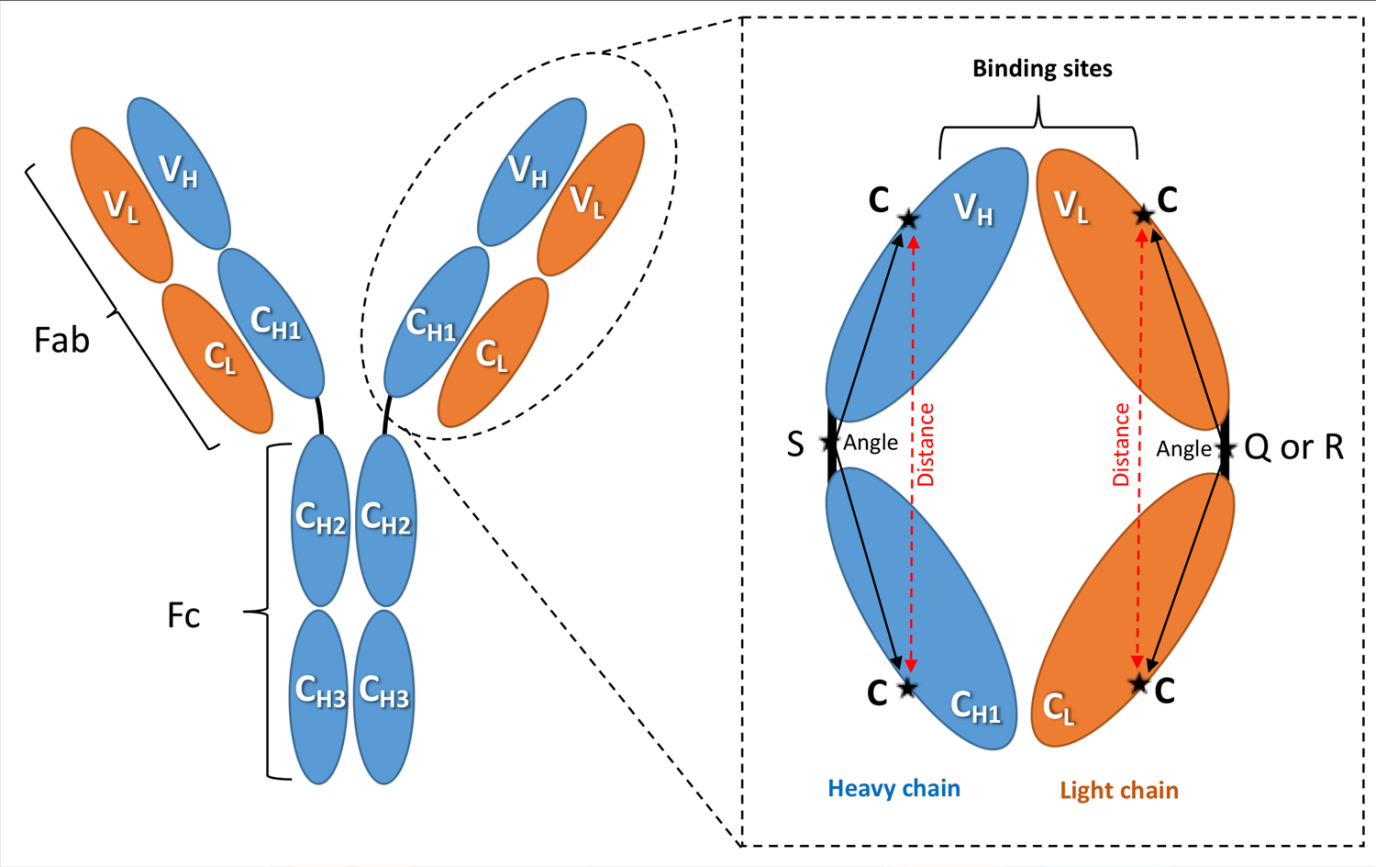
Angle measurements. The IgG antibody can be dissected into three fragments: two identical antigen-binding fragments (Fabs) that each contain the first two domains of the heavy (V_H_ and C_H1_) and light (V_L_ and C_L_) chains, and one crystallisable region fragment (Fc) consisting of two monomers, each comprising C_H2_ and C_H3_ domains. The amplified Fab region illustrates the amino acid positions used to calculate the domain orientational changes. The selected amino acids are denoted as C: cysteine, S: serine, Q: glutamine (in λ light chains), and R: arginine (in kappa light chains).

Analysis of the heavy and light chain orientations of the anti-protein Fabs (Scheme 1) revealed high angle changes in four of the couples, as summarised in Table 2 (full results are provided in the Supplementary Materials).

**Table 2:** Binding conformational change classes.

PDB structures	Magnitude of average change in angle	Class for binding conformational change
3G6A/3G6D	37°	B1
3HMW/3HMX	16°	B1
3EO9/3EOA	25°	B1
3EO9/3EOB	25°	B1
1MLB/1MLC	4°	B2
1DQQ/1DQJ	4°	B2
5BVJ/5BVP	2°	B2
2FJF/2FJG	3°	B3

Based on these movements, three different classes of behaviour can be identified, denoted B1-3. In class B1, the binding results in large conformational changes that can be pictured as a changing aspect ratio of the diamond-like shape of the Fab. In particular, these changes in angle come from a reduction in the angle between the heavy chains and an increase in that between the light chains. The angle changes were additionally confirmed by corresponding distance changes between key cysteine residues on each domain (see Scheme 1). The linker-linker distance of this B1 class has also increased in the bound form when compared to their free counterparts.

In contrast, the other two classes of behaviour (B2 and B3 in Table 2) show relatively small changes to the angles. The distinction between B2 and B3 will be explained in the following sections.

### Conformational changes and root-mean-square-deviation (RMSD)

The conformational changes were determined by calculating the total RMSD between the antibody-antigen complex and the antibody-free counterparts. This is obtained from the optimal overlap of the entire Fab. From these overlapped structures, the RMSD can also be calculated for each domain (note that the individual domains are not separately optimised here). As summarised in Table 3, all the anti-protein couples showed more overall movement upon binding in the heavy chains. In addition, the constant domains (C_H_ and C_L_) of these Fabs tend to have bigger RMSD values than the variable domains (V_H_ and V_L_). The shape of the antibody binding surface can be optimised through movements of the constant domains relative to the variable domains and to each other to generate a suitable V_H_-V_L_ relative orientation^18–20^.

**Table 3:** Trends in RMSD.

Light chain > heavy chain	None
Light chain < heavy chain	All 8 couples
**Light chain**	
CL < VL	2FJF/2FJG 3EOA/3EO9 3EOB/3EO9
CL > VL	Remaining 5 couples
**Heavy chain**	
CH < VH	2FJF/2FJG
CH > VH	Remaining 7 couples

The RMSD values of all PDB structures analysed here are listed in Supplementary Table S1.2.

The high movement of constant domains might also be correlated with signal propagation. We noticed these movements through analysis of an entire antibody in molecular dynamics simulations^21^. These movements are logically expected to be more in the heavy chain when compared to light chains, since the Fab-to-Fc allosteric movements occur in these heavy chains and through the main Fab-Fc linker. We explore prospective allosteric movements in the following section.

### Conformational changes and root-mean-square-fluctuations (RMSF)

**Figure 1:**
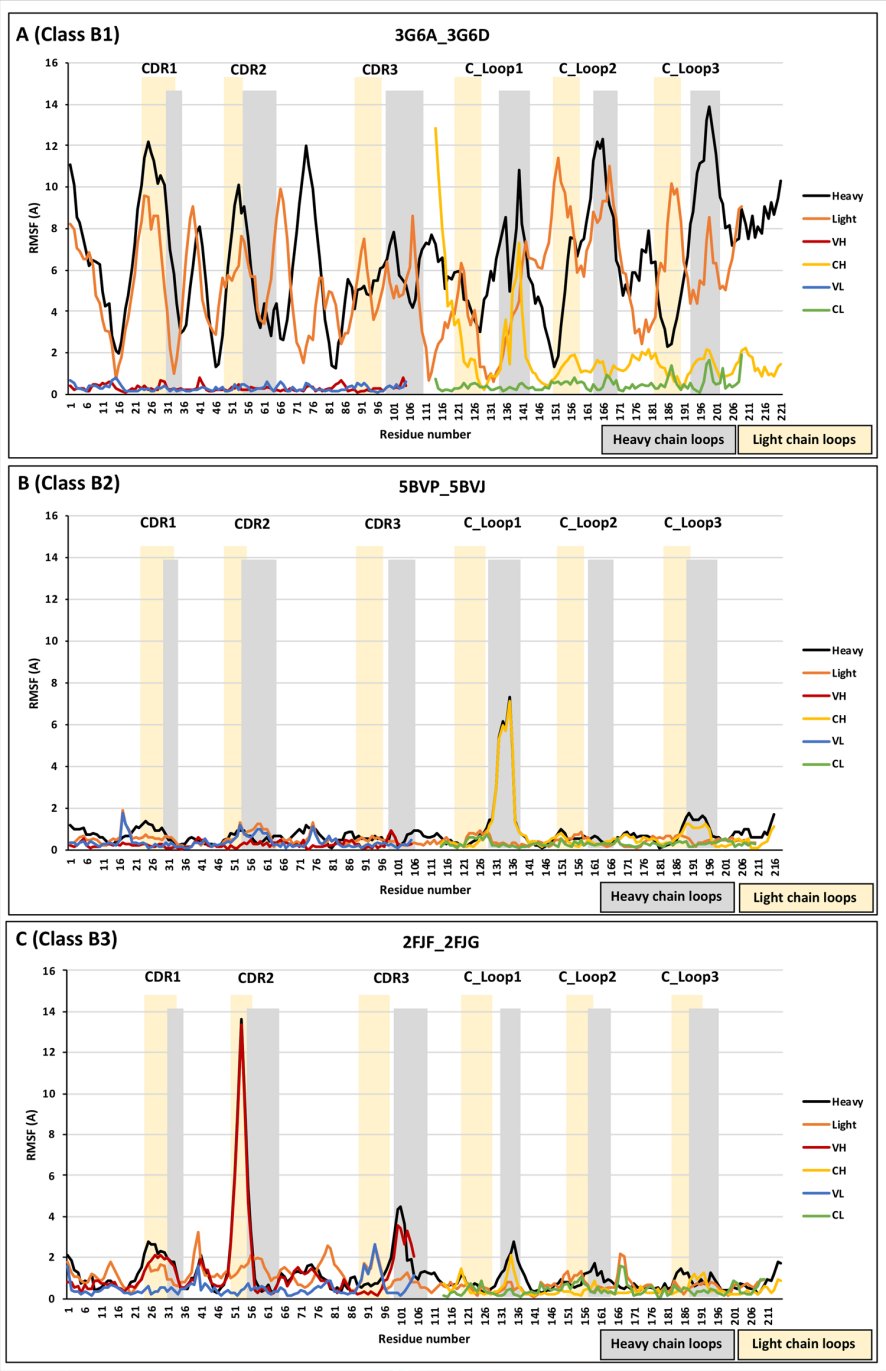
RMSF to illustrate the three antigen binding classifications reported in Table 2. (**A**) 3G6A/3G6D from class B1; (**B**) 5BVP/5BVJ from class B2; and (**C**) 2FJF/2FJG from class B3.

The RMSF, reporting deviations in the backbone position of each residue between the free and antigen bound antibodies, are reported in Fig. 1. Each of the binding conformational change categories from Table 2 is illustrated by one couple. In Fig. 1, we show the RMSF for the entire heavy (light) chain obtained from its optimal overall. We also show the RMSF calculated for each domain, using the optimal overlap of each domain separately. In this way we are able to distinguish which parts of the structure change and in what way. To obtain the data presented in Fig. 1, two crystal structures were compared: one as a free antibody and the second as an antibody/antigen complex. The RMSF was analysed for each amino acid position in the two compared crystal structures. This comparison was performed on the total chain (heavy and light) and independently on their specific domains (VH, CH, VL, and CL). The x-axis in Fig. 1 represents the amino acid positions of either the heavy or light chain running from the N- to C-terminus, allowing them to be compared on the same figure. Heavy chain loops are highlighted in grey and light chain loops are highlighted in yellow. The three loops in the variable domains are named CDR1-3, and he three loops in the constant domains (CH and CL) are named C_Loop1-3, according to their location throughout the sequence from the N-terminal to the C-terminal of the entire chain. These six loops were respectively shaded as grey and yellow for the heavy and light chains.

The top panel in Fig. 1 shows the RMSF for the 3G6A/3G6D couple in class B1. Both the heavy and light chains display significant RMSF values across the Fab sequence, dominated by the large conformational changes reported in Table 2 (recall that these RMSF are calculated from the optimal overlaps of the full chains between the structures in the couple). The loop structure of the heavy and light chains along their sequences is reflected in the RMSF pattern as might be expected. We also show the RMSF calculated from the optimal overlap of each domain separately. These RMSF values tend to be much lower, demonstrating a hinge-like movement between fairly rigid domains induced by the antigen adsorption. This is in line with the analysis of the angular changes between the domains in the Fab reported above.

The C_H1_ domain of class B1 by itself also displays large RMSF values in the region of the C_Loop1, as well as movements in its CDRs. The latter could be attributed to the binding between the antibody and antigen, while the former is probably associated with an allosteric signal propagation to the Fc region. As seen in the Supplementary Materials, the other couples in the B1 class (Table 2) also display very similar behaviour, with large RMSF overall and the CH domain displaying large values in the vicinity of the C_Loop1 and CDR3.

**Figure 2:**
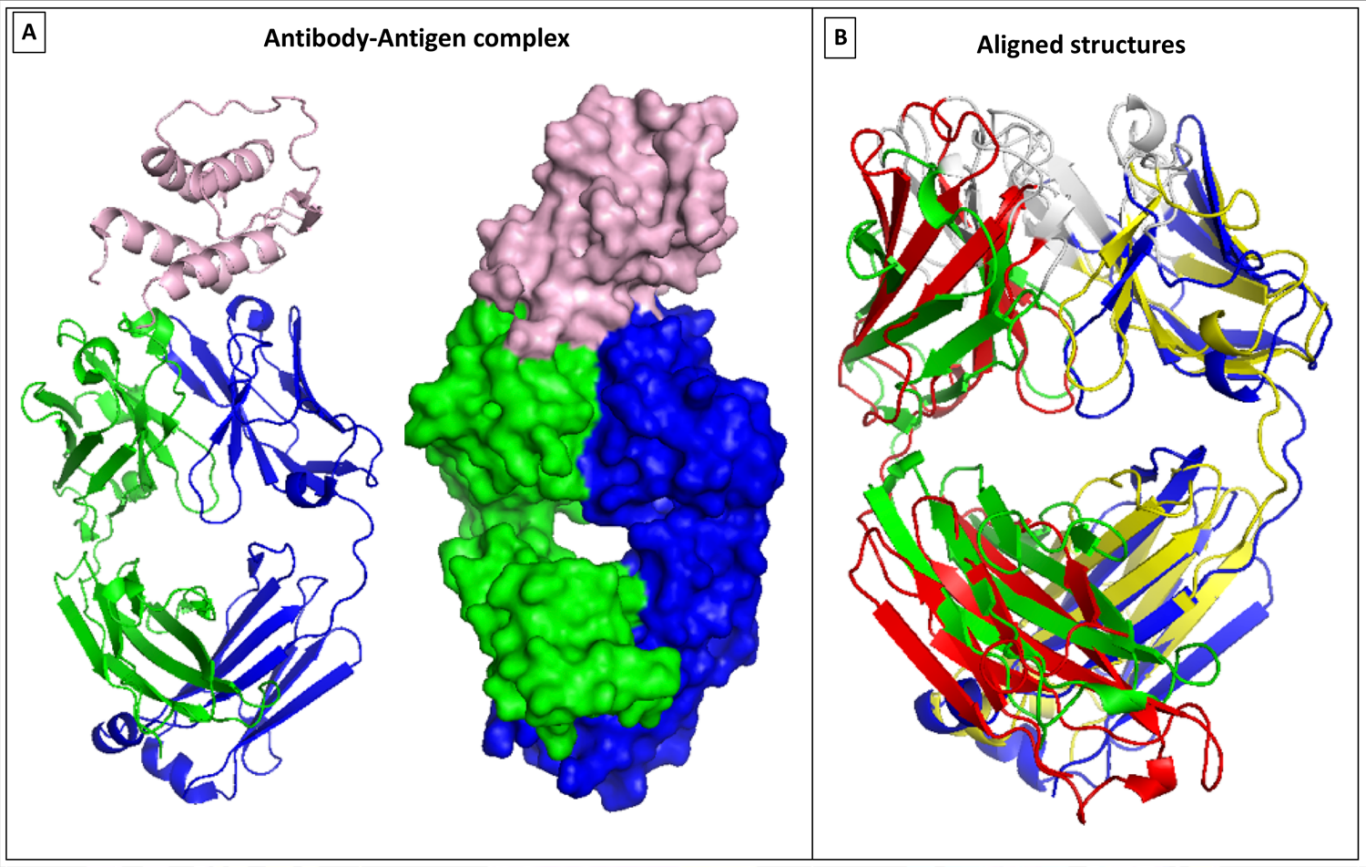
Category B1 conformational changes observed in 3G6A/3G6D, using VMD 1.9.3^22^. (**A**) Illustrates the bound configuration of the antigen–antibody with the heavy chain in blue and light chain in green, and the antigen white. (**B**) Displays the structural overlap of the entire Fab between the bound (blue/green) and free (red/yellow) structures.

Figure 2 illustrates the relative domain conformational changes induced in category B1. The overlap between 3G6A and 3G6D are shown in cartoon representation to clarify the changes to the diamond-like Fab structure when the antigen protein binds in the vicinity of the groove between the VL and VH domains (the left panel in Fig. 2 illustrates the binding configuration, with the right panel showing the overlaps). It seems intuitive that this distortion of the Fab conformation will allow better interaction between the antigen and antibody, permitting more inter-residue interactions between the biomolecules.

**Figure 3:**
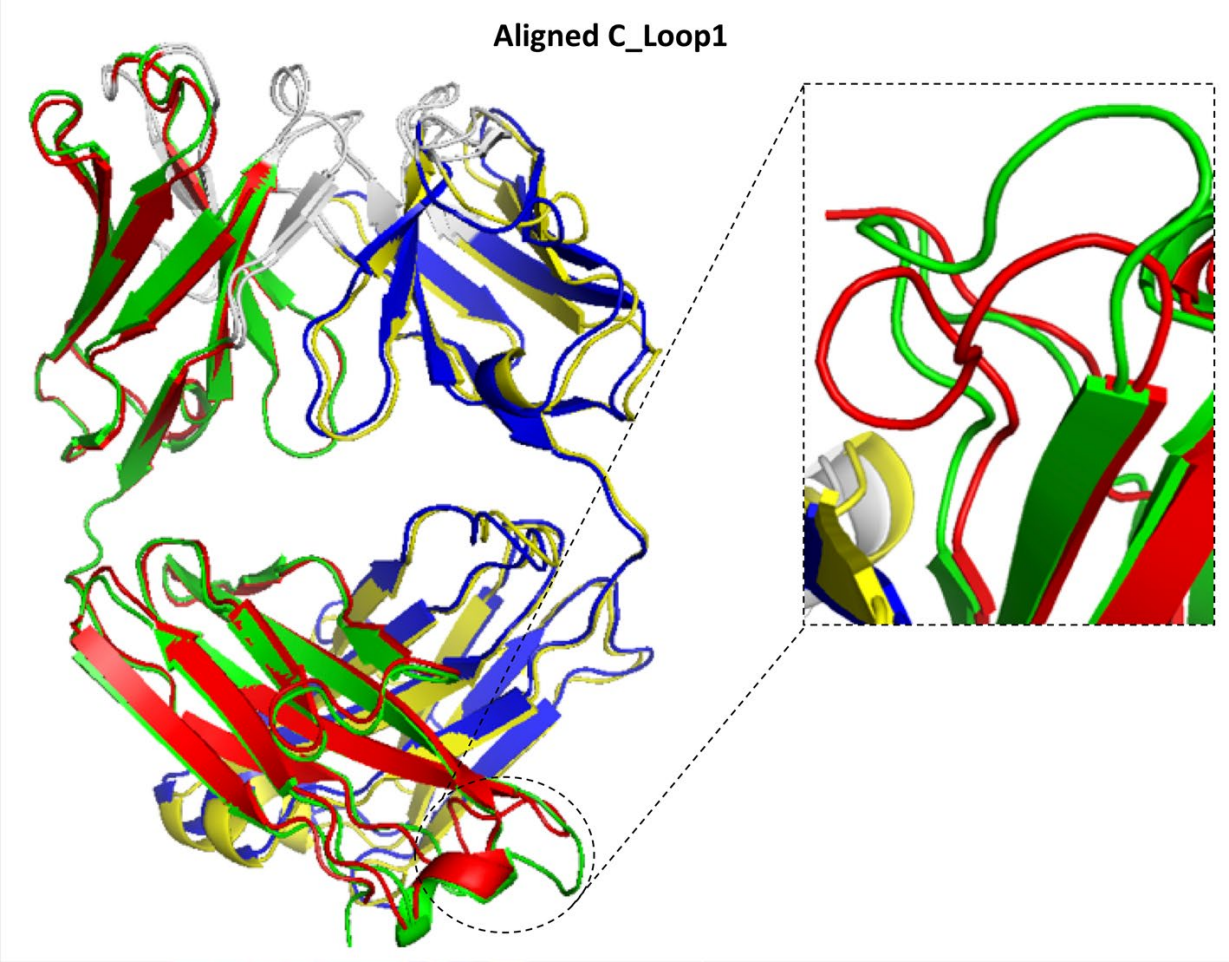
B2 Conformational changes—focus on the C_Loop1 heavy chain movement in 5BVP/5BVJ, using VMD 1.9.3^22^. This figure illustrates the deviation in C_Loop1 between the bound versus the free form of the Fabs (5BVP/5BVJ).

Returning to Fig. 1, the middle panel shows the RMSF results for an exemplar of class B2 for binding conformational changes. In contrast to B1, in the B2 class we did not find large wholesale RMSF values when we overlapped the light and heavy chains in their entirety, because there are no large relative orientational changes to the domains (see Table 2). In other words, the diamond-like structure of the Fab remains largely unchanged upon binding with the antigen in this category (Fig. 3). Surprisingly, however, we did find that there is again a large change in the C_Loop1 region of the CH domain. Again, this might indicate a possible allosteric change propagated to the Fc region of the antibody. This behaviour is replicated in the other couples in the B2 category (see Supplementary Materials), along with varying degree of change to the CDR regions induced by the antigen binding. The change in the C_Loop1 region of the 5BVP structure is illustrated in Fig. 3.

**Figure 4:**
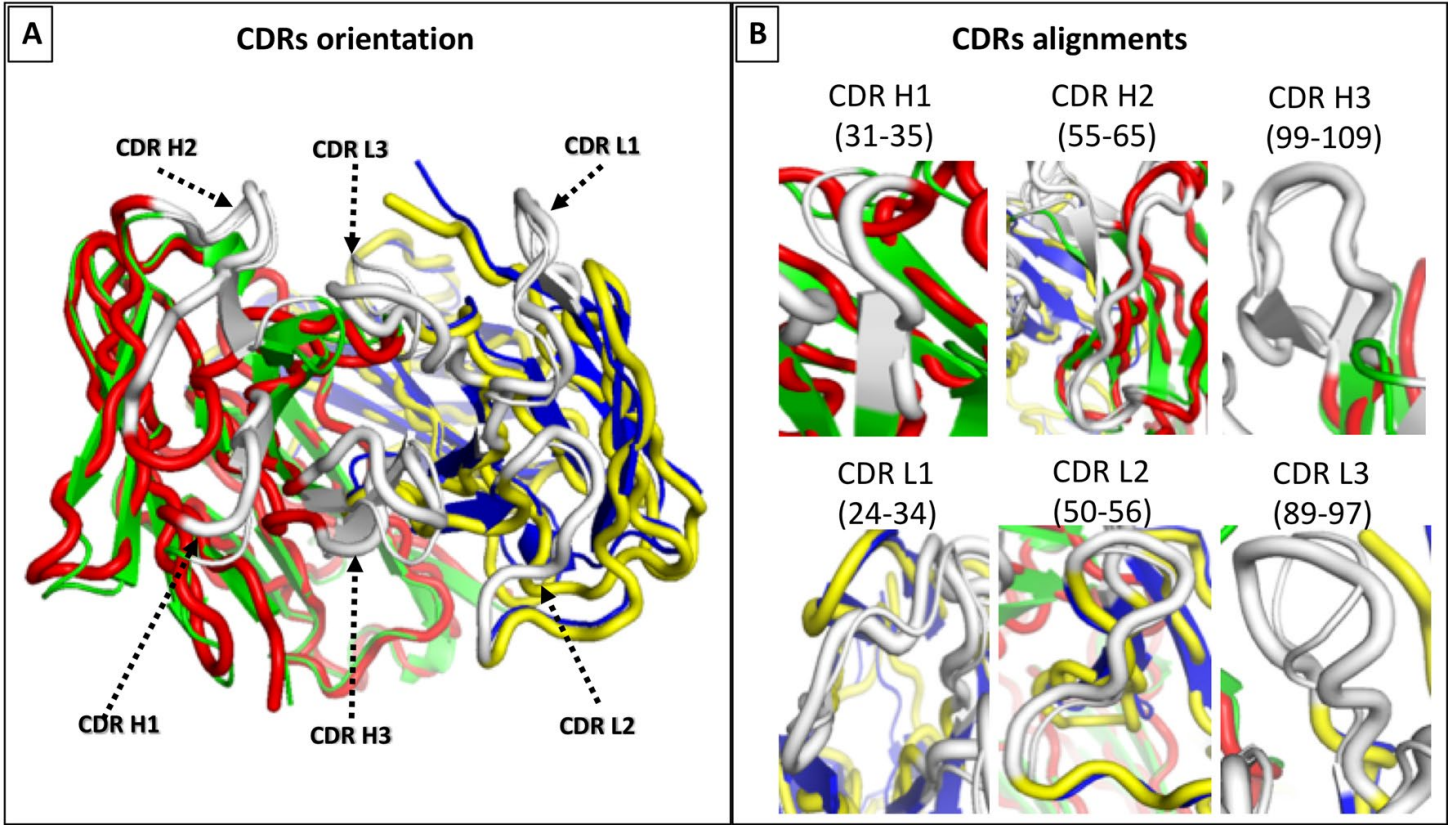
Category B3 conformational changes observed in 2FJF/2FJG, using VMD 1.9.3^22^. (**A**) Illustrates the binding surface of the antibody along with the CDRs distribution across the surface. (**B**) displays the CDRs alignment comparison between the bound (blue/green) and free (red/yellow) structures. The CDRs are shown in white, with the bound structures thinner that the unbound ones for clarity of comparison.

Finally, we discuss the 2FJF/2FJG couple from class B3 (Fig. 4). Unlike the previous examples, this antibody shows no significant change in the C_Loop1 region upon binding to its target (Fig. 1). Instead, we see a very strong change occurring at the binding site only, with prominent changes in the VH domain at CDR2 and to a lesser extent at CDR3; no other large scale alterations to the Fab structure are observed. Close-up views of the changes at the CDRs are illustrated in Fig. 4. This different behaviour suggests that this antibody does not require an allosteric signal propagation. Indeed, it is a phage-derived antibody from a synthetic library rather than a naturally occurring antibody (Table 1), and it would seem that while it is selected for its binding affinity, its inability to propagate an allosteric signal upon binding has not been part of the selection process.

This movement in CDR-H2 was observed by the researchers who crystalised 2FJF and 2FJG, and was attributed to the adoption of a highly unusual and non-canonical conformation of CDR-H2 following the antigen binding^23^. This was caused by a tight packing of CDR-H2 against CDR-H1 and a section of the antibody that is part of the framework 3 region. This drastic conformational change of CDR-H2 was facilitated by three glycines at positions 50, 54, and 55, which are likely to provide CDR-H2 the flexibility required to show such a significant conformational change. In contrast, the same authors found CDR-H2 of the free antibody (2FJF) is in a very different position and packs against CDR-L3 and CDR-H3, following the commonly observed canonical conformation. The high movement of CDR-H2 of 2FJG when compared to 2FJF was observed in our RMSD analysis of this pair, as demonstrated in Fig. 1C.

## Discussion and conclusions

We have shown how straight-forward analyses of crystal structures can help identify possible allosteric signalling pathways caused by antibody-protein antigen binding. The analyses focus on backbone structural changes, and avoid the necessity for expensive simulations. The assumption is made that the crystal contacts in the PDB structures do not strongly affect the backbone structure; this assumption can be explored further in future work.

In this work, we have compared the crystallised Fab structures of 8 couples, each of which has a free IgG antibody and the same antibody bound to its protein antigen target. In so doing, we identify three classes of behaviour:

- B1: The binding causes a deformation of the diamond-like Fab structure, with the four domains (V_H_, V_L_, C_H1_ and C_L_) maintaining their individual structures but changing relative orientation by hinging at the linker regions between them. This category displays a further prominent change in the C_Loop_1_ region indicating an allosteric signal propagation.
- B2: The binding does not deform the overall diamond-like Fab structure, but still shows the prominent C_Loop_1_ deformation as in B1.
- B3: The binding only causes changes at the CDR regions directly interacting with the antigen, and no obvious allosteric signalling pathway is apparent.

The class B3 results were surprising, however, it is based on only one protein antigen example, and this is derived from a phage display for its binding affinity. Nevertheless, it holds a cautionary lesson for the development of therapeutics from small antibody fragments; it is not sufficient to optimise binding locally at the CDRs if ultimately one requires an Fc effector response to be initiated by the binding to the antigen. In fact, we identified more antibodies of class B3, which were developed against hapten-antigens.

Our results for class B1 and B2 are in line with observations made elsewhere. Sela-Culang et al.^16^ showed that C_Loop_1_ is the highest moving loop, deviating even more than CDRH3 when binding the antigen. Various other studies have correlated the C_H1_ domain to changes in binding, because it was recognised as the only region with sequence diversity between the antibodies being examined^22–24^. It has been suggested that the C_Loop_1_, linking the heavy and light chains, is intrinsically disordered and involved in complement binding^16^. Furthermore, the C_Loop_1_ is part of the interface with the C_L_ domain, and occasionally connected to the C_L_ domain through a disulphide bond, thereby having a potential effect on the C_H1_-C_L_ relative orientation. Moreover, this loop is close to the main hinge region, and a conformational change in this loop may affect the relative orientation of the C_H1_ domain *versus* the entire Fc, thus influencing the overall effector function of the Fc region^17,25^. Therefore, this loop can be highly prone to movement to fulfil the required structural changes upon binding.

The higher movements of the constant domains compared to the variable domains observed in B1 (and to some extent in B2) have been discussed elsewhere with regard to antigen binding affinity. For instance, two human monoclonal antibodies having similar variable domains, but different constant domains, were shown to bind their target with significantly different affinities^22^. Furthermore, comparing the binding affinity of Fabs to smaller versions of variable fragments (Fv) have also revealed a large difference in affinity^26^. In fact, the binding epitopes of two anti-HIV1 IgG_1_ and IgA_2_ antibodies with identical variable domains are only partially overlapping^27^. Similarly, analysis of trastuzumab, crenezumab, and pertuzumab recombinant models have suggested that antibodies with the same variable domains, but different constant domains, have significantly different antigen binding and affinity^28,29^.

In summary, the binding of antibodies to their protein antigens can induce strong structural deformations that can propagate through the heavy chain to the Fc regions, potentially triggering an effector response, and in this the C_H_ domain and its C_Loop1 plays a prominent role. In addition, changes in the constant domains can also be important to the antigen affinity. However, antigen binding does not universally induce these effects, and this implies that careful assessment of binding-induced structural changes must be undertaken when developing new therapeutics.

## Methods

### Antibody selection

The structures of Fabs from several IgG antibodies, crystallised as both free and antigen protein-complexed formats and reported in the same articles, were retrieved from the PDB. Only structures with acceptable resolution (< ~ 3 Å) were included in the analysis to allow a confident determination of the molecular interactions and structures^9,30,31^. A total of 15 crystal PDB structures against protein antigens (4 mouse and 11 full human) were identified and selected (Supplementary Table S1.1). Free antibody structures were considered similar if their chains (heavy and light) shared 100% sequence identity, and have similar sequence to the antigen bound form. For instance, one crystal structure (PDB entry 2FJF) contains 12 Fabs in the asymmetric unit to form a single dodecameric complex. We have selected one Fab of the antibody free form to be compared with the antigen bound form (PDB entry 2AJG), since the authors of the PDB entry report that these 12 structures share the same crucial features^23^. Similarly, with other cases of multiple structures in the PDB entry, we select one on the understanding that the other structures are the same in all essential features.

While preparing the retrieved PDB files, all amino acid positions were sequentially numbered to avoid missing or duplicating residue positions, and to ensure that calculations to compare structures gave correct measurements. A few sequences (5GGV, 5GGU, 3NFP and 3NFS) were omitted from the study due to the fact that the reported polypeptide chains were not continuous, so that the remaining 15 structures studied allowed detailed analyses.

The selected structures were organised into 8 couples as detailed in Table 1, and for each couple, two crystal structures were compared: one for the free antibody and the second for the antibody/antigen complex. The analyses included: alignment of the CDRs; alignment of the two crystal structures to compare deviation in the antigen binding areas; quantification of the deviation between the structures overall, by light and heavy chain conserved and variable regions, and on an individual amino acid basis; and quantification of the relative orientation of the heavy and light domains. The three loops in the variable domains were denoted CDRs 1–3, and the three loops in the constant domains (CH and CL) were named C_Loop 1–3, according to their location throughout the sequence from the N-terminal to the C-terminal of the entire chain.

The PDB Fab sequences were retrieved and analysed using BioEdit Sequence Alignment Editor, version 7.2.5^32^. ClustalW Multiple alignment was used to align sequences of the same type (heavy or light chains) and origin (human or mouse). All the gathered and analysed sequences are listed in Supplementary Table S3.1–S3.5. The six CDRs of the Fab fragments were defined using the Kabat numbering system^33^.

Note that in our analyses, we assume that the crystal contacts in the PDB structures will have negligible effects on results. This is justified, because we analyse structural changes in terms of backbone movements rather than side-chains, which are most likely affected by crystal formation.

### RMSD and RMSF

RMSD is a commonly used tool that quantifies the structural overlap of protein structures, such as different conformations of the same protein^34^. For this study, the RMSD is defined as

1 $$ RMSD = \sqrt {\frac{{\mathop \sum \nolimits_{i = 1}^{N} \left| {\vec{r}_{i,A} - \vec{r}_{i,F} } \right|^{2} }}{N}} $$

where N is the number of (backbone) atoms in the protein structure and $\vec{r}_{i,A}$, $\vec{r}_{i,F}$ is the position of the i-th atom in the antigen-bound (A) and free (F) Fab structure. In practice, to calculate the RMSD, the two protein structures to be compared are initially treated as rigid bodies that are overlapped (aligned) using only translations and rotations. The RMSDs of the backbone atoms only were calculated for each couple for the entire antibody chain (residues 1–218) as well as particular regions, namely: variable (residues 1–105), linker (106–113) and constant (114–218) domains. The side chains of the residues were excluded from the RMSD calculations, since differences in their orientations could introduce additional noise. Moreover, we are interested in global structural changes, not the local alterations in side chain orientation.

Another beneficial tool to track structural changes in proteins is denoted RMSF, where the RMSD is calculated and reported for each residue in the compared structure. It is regularly referred to as “fluctuation” because it measures an amplitude of residue movement (fluctuation) from the compared position in the aligned structures. Although usually measured for dynamic structures, for static structures it provides the local RMSD calculated for each residue. Therefore, the traditional RMSD gives one number reflecting the average difference between the entire protein chains being compared, while RMSF “divides” this difference and reports it per residue. Consequently, it is easy to track the residues (and so sequence parts) which do not change conformation and the residues (sequence parts) which are the most responsible for the observed conformational alterations upon antigen binding. The RMSDs are calculated using the VMD^35^ built-in tool while RMSFs are calculated using a Tcl script executed from the VMD Tk console. The unit for both is Å (10^−10^ m).

### Variable and constant domain orientation

The relative orientation of the variable heavy and light (V_H_ and V_L_) *versus* conserved heavy and light (C_H1_ and C_L_) domains was quantified to complement the RMSD/RMSF analyses. One conserved cysteine residue (Cys) was selected in each of the V_L_, C_L_, V_H_, and C_H1_ domains (Scheme 1). In addition, one conserved amino acid in each of the V_L_/C_L_ and V_H_/C_H1_ linkers was also selected; these are Serine (Ser), Arginine (Arg), and Glutamine (Gln) in the heavy, light (K), and light (λ) chains, respectively (Scheme 1). The six amino acids thus selected in each Fab are highlighted in turquoise in the full sequence file (Supplementary S3). The angles of the heavy (C-S-C) and light (C-Q-C or C-R-C) chains were measured by PyMOL (The PyMOL Molecular Graphics System, Version 1.7.4 Schrödinger, LLC.) and confirmed by VMD^35^. The distances from the two Cys residues in the heavy and light chains were additionally measured by PyMOL and VMD.

## Supplementary information

Supplementary file1
