# Supplementary material for: Antibody-protein binding and conformational changes: identifying allosteric signalling pathways to engineer a better effector response
Source: Sci Rep. 2020 Aug 13;10:13696. doi: 10.1038/s41598-020-70680-0 (PMC7426963; doi:10.1038/s41598-020-70680-0)
Supplement: Supplementary file 1 — Supplementary file1 [file 41598_2020_70680_MOESM1_ESM.pdf]

Antibody-protein binding and conformational changes: identifying allosteric signalling pathways to engineer a better effector response

Mohammed M. Al Qaraghuli[1,2,*], Karina Kubiak-Ossowska[1,3], Valerie A. Ferro[4], and Paul A. Mulheran[1]

1 Department of Chemical and Process Engineering, University of Strathclyde, Glasgow, UK, G1 1XJ.

2 SiMologics Ltd. The Enterprise Hub, Level 6 Graham Hills Building, 50 Richmond Street, Glasgow, UK, G1 1XP.

3 Department of Physics, University of Strathclyde, Glasgow, UK, G4 0NG

4 Strathclyde Institute of Pharmacy and Biomedical Sciences, University of Strathclyde, 161 Cathedral Street, Glasgow, UK, G4 0RE.

* Correspondence: Email: mohammed.al-qaraghuli@strath.ac.uk; Tel.: +44 (0)141 548 2176

Supplementary Data

Supplementary S1: Structures selection and classification 3
Table S1.1: Details of selected crystal structures 3
Table S1.2: RMSD measurements 7
Supplementary S2: Antibodies structural analyses 9
S.2.1 (1MLB vs 1MLC), mouse: 10
S.2.2 (1DQQ vs 1DQJ), mouse: 12
S.2.3 (3G6A vs 3G6D), human: 14
S.2.4 (2FJF vs 2FJG), human: 16
S.2.5 (3HMX vs 3HMW), human: 18
S.2.6 (3EOA_3EO9), human: 20
S.2.7 (3EOB vs 3EO9), human: 22
S.2.8 (5BVP vs 5BVJ), human: 24
Supplementary S3: Sequences Alignment 26
Table S3.1 Protein_Human_Heavy chain_11 sequences 27
Table S3.2 Protein_Human_Light chain_Kappa_9 sequences 28
Table S3.3 Protein_Human_Light_Lambda_2 sequences 29
Table S3.4 Protein_Mouse_Heavy chain_4 sequences 30
Table S3.5 Protein_Mouse_Light_Kappa_4 sequences 31
Supplementary S4: Angles and distances 32

Supplementary S1: Structures selection and classification

Table S1.1: Details of selected crystal structures

PDB number	PDB ID	Source (format)	Crystal structure description	Target (Molecular weight-Da)	Crystal form	Clone name (heavy/light)	Genetic class	References
1	1MLC	Mus musculus	Monoclonal antibody fab d44.1 raised against chicken egg-white lysozyme complexed with lysozyme	Chicken egg-white lysozyme (14313.14)	Antigen-antibody	D44.1	(IgG1-Kappa)	(Braden et al. 1994)
2	1MLB	Mus musculus	Monoclonal antibody fab d44.1 raised against chicken egg-white lysozyme	--	Antibody	D44.1	(IgG1-Kappa)	(Braden et al. 1994)
3	1DQJ	Mus musculus	Crystal structure of the anti-lysozyme antibody hyhel-63 complexed with hen egg white lysozyme	Hen egg white lysozyme (14313.14)	Antigen-antibody	HYHEL-63	(IgG2a-Kappa)	(Y. Li et al. 2000)
4	1DQQ	Mus musculus	Crystal structure of anti-lysozyme antibody hyhel-63	--	Antibody	HYHEL-63	(IgG2a-Kappa)	(Y. Li et al. 2000)

5	3G6D	Homo sapiens	Crystal structure of the complex between CNTO607 Fab and IL-13	IL-13 (12475.59)	Antigen-antibody	CNTO607	(IgG1-Lambda)	(Teplyakov et al. 2009)
6	3G6A	Homo sapiens	Crystal structure of anti-IL-13 antibody CNTO607	--	Antibody	CNTO607	(IgG1-Lambda)	(Teplyakov et al. 2009)
7	2FJG	Homo sapiens	Structure of the G6 Fab, a phage derived Fab fragment, in complex with VEGF	VEGF-A (11932.65)	Antigen-antibody	G6	(IgG-Kappa)	(Fuh et al. 2006)
8	2FJF	Homo sapiens	Structure of the G6 Fab, a phage derived VEGF binding Fab	--	Antibody	G6	(IgG-Kappa)	(Fuh et al. 2006)

9	3HMX	Homo sapiens	Crystal structure of ustekinumab FAB/IL-12 complex	IL-12 (34696.96)	Antigen-antibody	Ustekinumab	(IgG1-Kappa)	(Luo et al. 2010)
10	3HMW	Homo sapiens	Crystal structure of ustekinumab FAB	--	Antibody	Ustekinumab	(IgG1-Kappa)	(Luo et al. 2010)
11	3EOA	Homo sapiens	Crystal structure the Fab fragment of Efalizumab in complex with LFA-1 I domain, Form I	Lymphocyte function-associated antigen 1 (20650.70)	Antigen-antibody	Efalizumab	(IgG1-Kappa)	(S. Li et al. 2009)
12	3EOB	Homo sapiens	Crystal structure the Fab fragment of Efalizumab in complex with LFA-1 I domain, Form II	Lymphocyte function-associated antigen 1 (20650.70)	Antigen-antibody	Efalizumab	(IgG1-Kappa)	(S. Li et al. 2009)

13	3EO9	Homo sapiens	Crystal structure the Fab fragment of Efalizumab	--	Antibody	Efalizumab	(IgG1-Kappa)	(S. Li et al. 2009)
14	5BVP	Homo sapiens	The molecular mode of action and species specificity of canakinumab, a human monoclonal antibody neutralizing IL-1beta	IL-1beta (17376.87)	Antigen-antibody	Canakinumab	(IgG1-Kappa)	(Rondeau et al. 2015)
15	5BVJ	Homo sapiens	The molecular mode of action and species specificity of canakinumab, a human monoclonal antibody neutralizing IL-1beta	--	Antibody	Canakinumab	(IgG1-Kappa)	(Rondeau et al. 2015)

Table S1.2: RMSD measurements

PDB number	PDB ID	Resolution (Å)	PDB couples	Constant (resid 114-218)	Variable (resid 1-105)	Linker (resid 106-113)	Total RMSD (VMD)	RMSD trend (domain)	RMSD trend (chain)	Change in topography
1	1MLC	2.5	1mlbL vs 1mlcL	0.83	0.41	0.26	0.99	CL more	Light less	No
	1MLB	2.1	1mlbH vs 1mlcH	1.17	0.62	0.2	1.12	CH more		
2	1DQJ	2	1dqqL vs 1dqjL	0.44	0.35	0.15	0.67	CL more	Light less	Yes
	1DQQ	1.8	1dqqH vs 1dqjH	1.08	0.73	0.24	1.12	CH more		
3	3G6D	3.2	3g6dL vs 3g6aL	0.55	0.34	1.34	5.91	CL more	Light less	Yes
	3G6A	2.1	3g6dH vs 3g6aH	2.68	0.33	0.22	6.99	CH more		
4	2FJG	2.8	2fjfL vs 2fjgL	0.51	0.66	0.25	1.06	CL more	Light less	Yes
	2FJF	2.65	2fjfH vs 2fjgH	0.63	2.33	0.21	1.89	VH more		

5	3HMX	3	3hmxL vs 3hmwL	0.96	0.59	0.3	3.15	VL more	Light less	No
	3HMW	3	3hmxH vs 3hmwH	1.52	0.43	0.17	3.28	CH more		
6	3EOA	2.8	3eoaL vs 3eo9L	0.44	0.51	0.35	3.72	CL more	Light less	No
	3EOB	3.6	3eoaH vs 3eo9H	1.64	0.41	0.24	4.97	VH more		
7	3EO9	1.8	3eobL vs 3eo9L	0.5	0.54	0.41	3.64	CL more	Light less	No
			3eobH vs 3eo9H	1.63	0.47	0.31	4.89	VH more		
8	5BVP	2.2	5bvpL vs 5bvjL	0.5	0.48	0.2	0.6	CL more	Light less	No
	5BVJ	2	5bvpH vs 5bvjH	1.47	0.31	0.15	1.21	CH more		

Supplementary S2: Antibodies structural analyses

For each couple, two crystal structures were compared using VMD 1.9.3: one as free antibody and the second as antibody/antigen complex. The selected couples were of human (6) and mouse (2) origin. The analyses included A) Comparison of the binding sites surfaces between a free antibody and antibody/antigen crystal structures. B) Orientation of the six CDRs of the two crystal structures. C) Demonstration of the antibody/antigen complex as a backbone cartoon or overall surface. D) Alignment of the CDRs along with their amino acid positions, which were numbered sequentially. E) RMSF comparison of each amino acid position in the two compared crystal structures. This comparison was performed on the total chain (heavy and light) and independently on their specific domains (V_H, C_H, V_L, and C_L). Heavy chain loops were highlighted in grey and light chain loops were highlighted in yellow. The three loops in the variable domains were named CDR1-3, and the three loops in the constant domains (CH and CL) were named C_Loop1-3, according to their location throughout the sequence from the N-terminal to the C-terminal of the entire chain. F) Alignment of the two crystal structures to compare deviation in the loops.

S.2.1 (1MLB vs 1MLC), mouse:

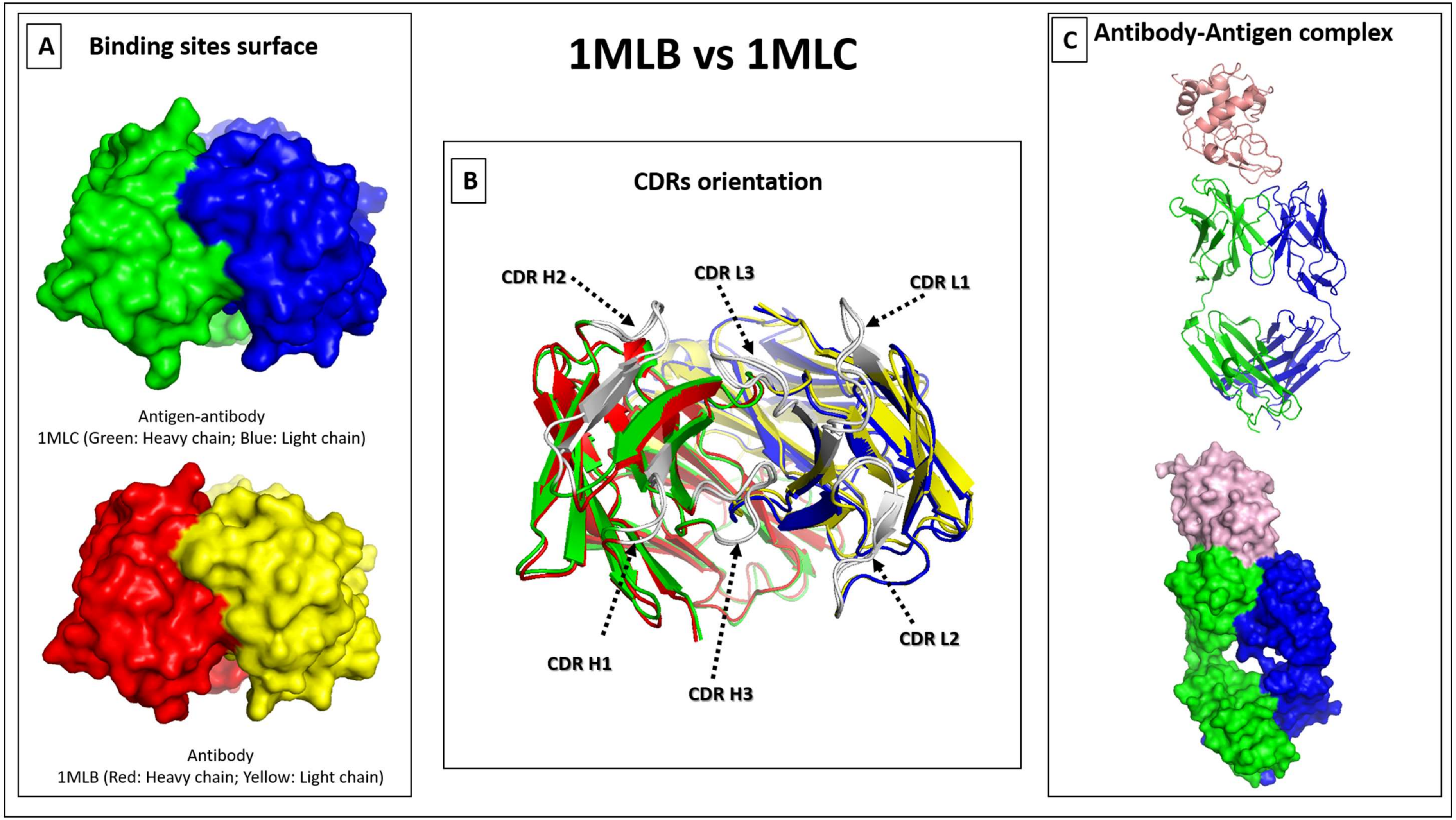

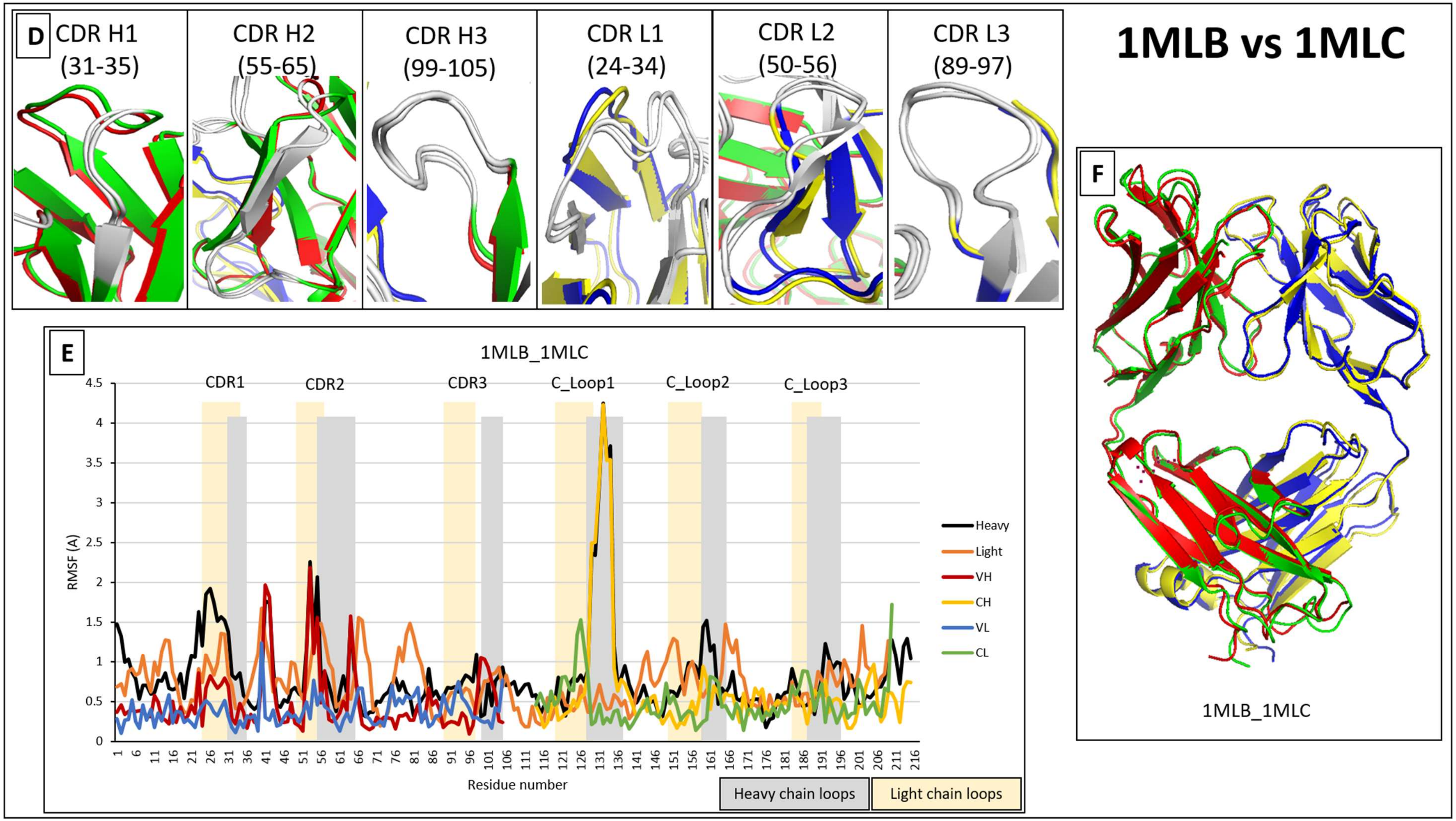
D
CDR H1
(31-35)
CDR H2
(55-65)
CDR H3
(99-105)
CDR L1
(24-34)
CDR L2
(50-56)
CDR L3
(89-97)
1MLB vs 1MLC
E
1MLB_1MLC
CDR1
CDR2
CDR3
C_Loop1
C_Loop2
C_Loop3
RMSF (A)
Residue number
Heavy
Light
VH
CH
VL
CL
Heavy chain loops
Light chain loops
F
1MLB_1MLC

S.2.2 (1DQQ vs 1DQJ), mouse:

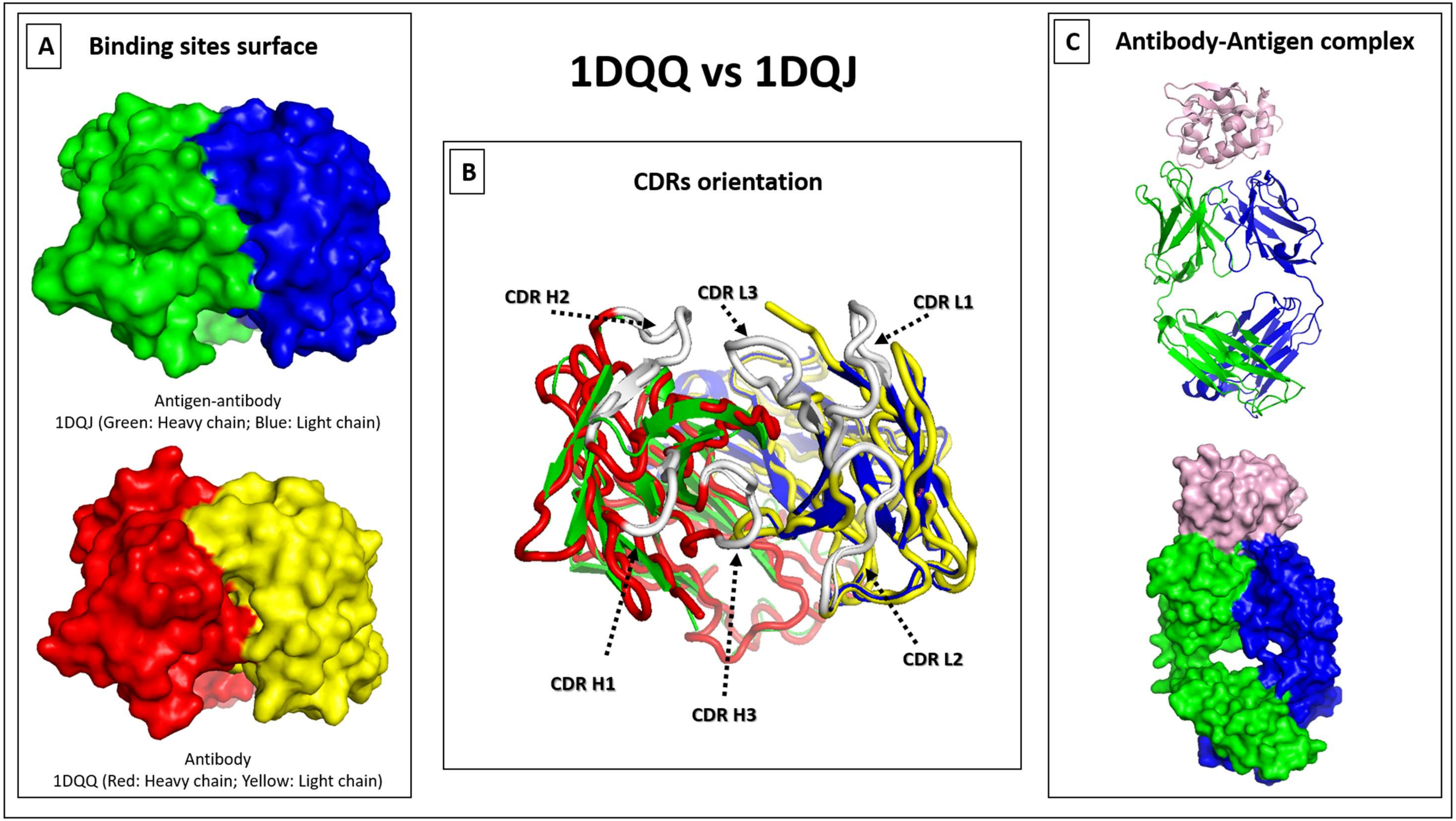

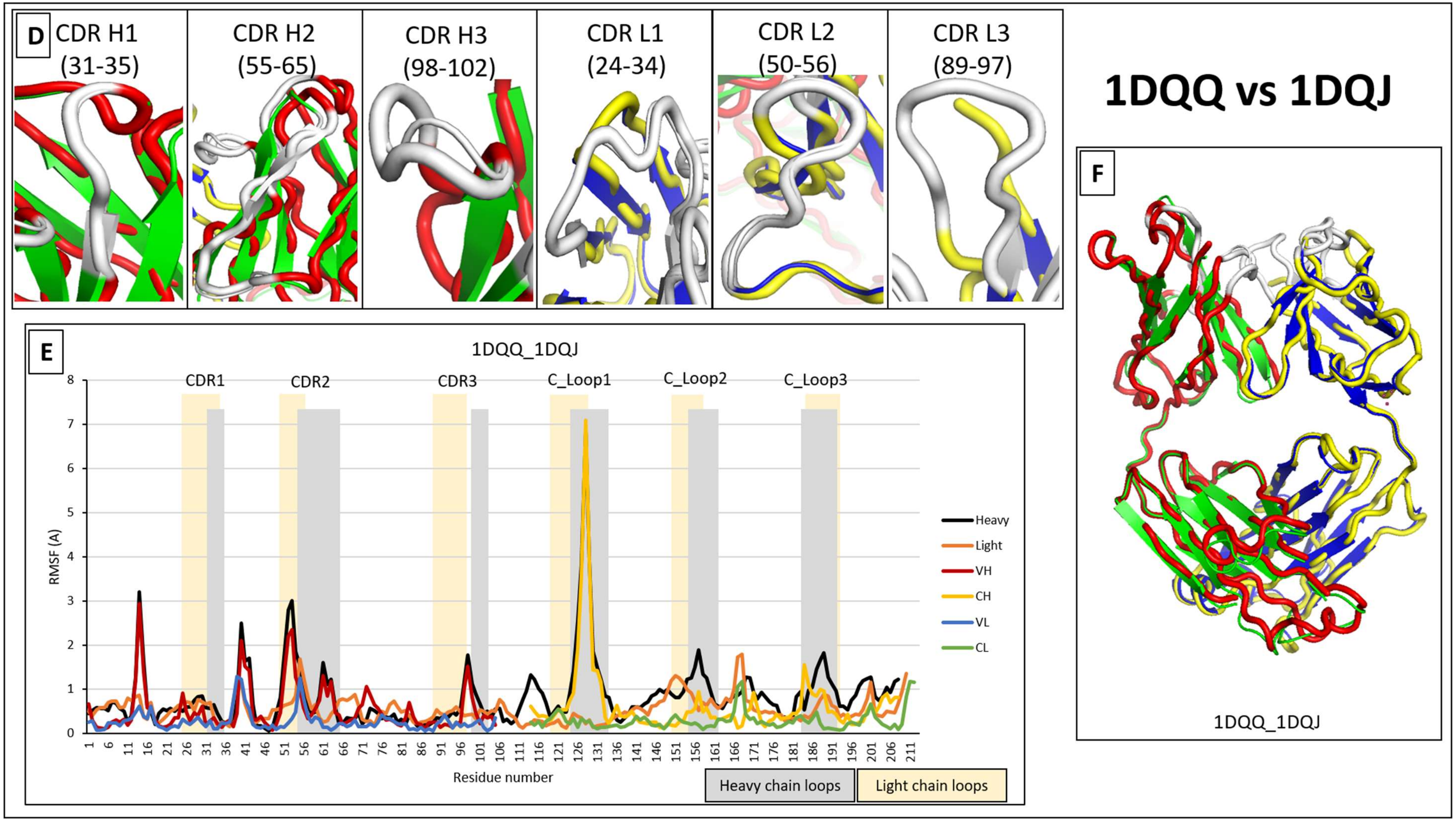
D
CDR H1 (31-35)
CDR H2 (55-65)
CDR H3 (98-102)
CDR L1 (24-34)
CDR L2 (50-56)
CDR L3 (89-97)
1DQQ vs 1DQJ
E
1DQQ_1DQJ
CDR1
CDR2
CDR3
C_Loop1
C_Loop2
C_Loop3
RMSF (A)
Residue number
Heavy
Light
VH
CH
VL
CL
Heavy chain loops
Light chain loops
F
1DQQ_1DQJ

S.2.3 (3G6A vs 3G6D), human:

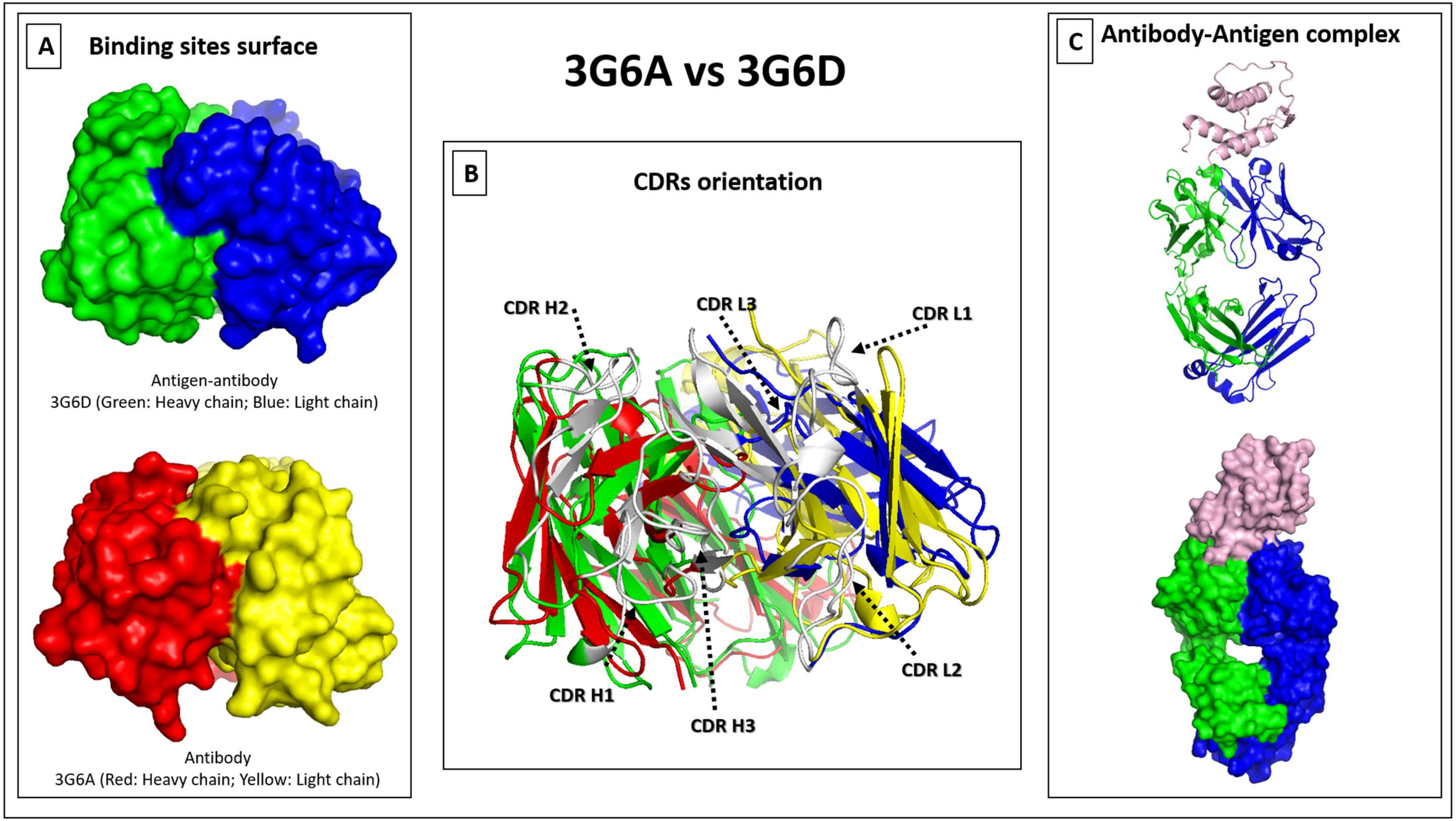

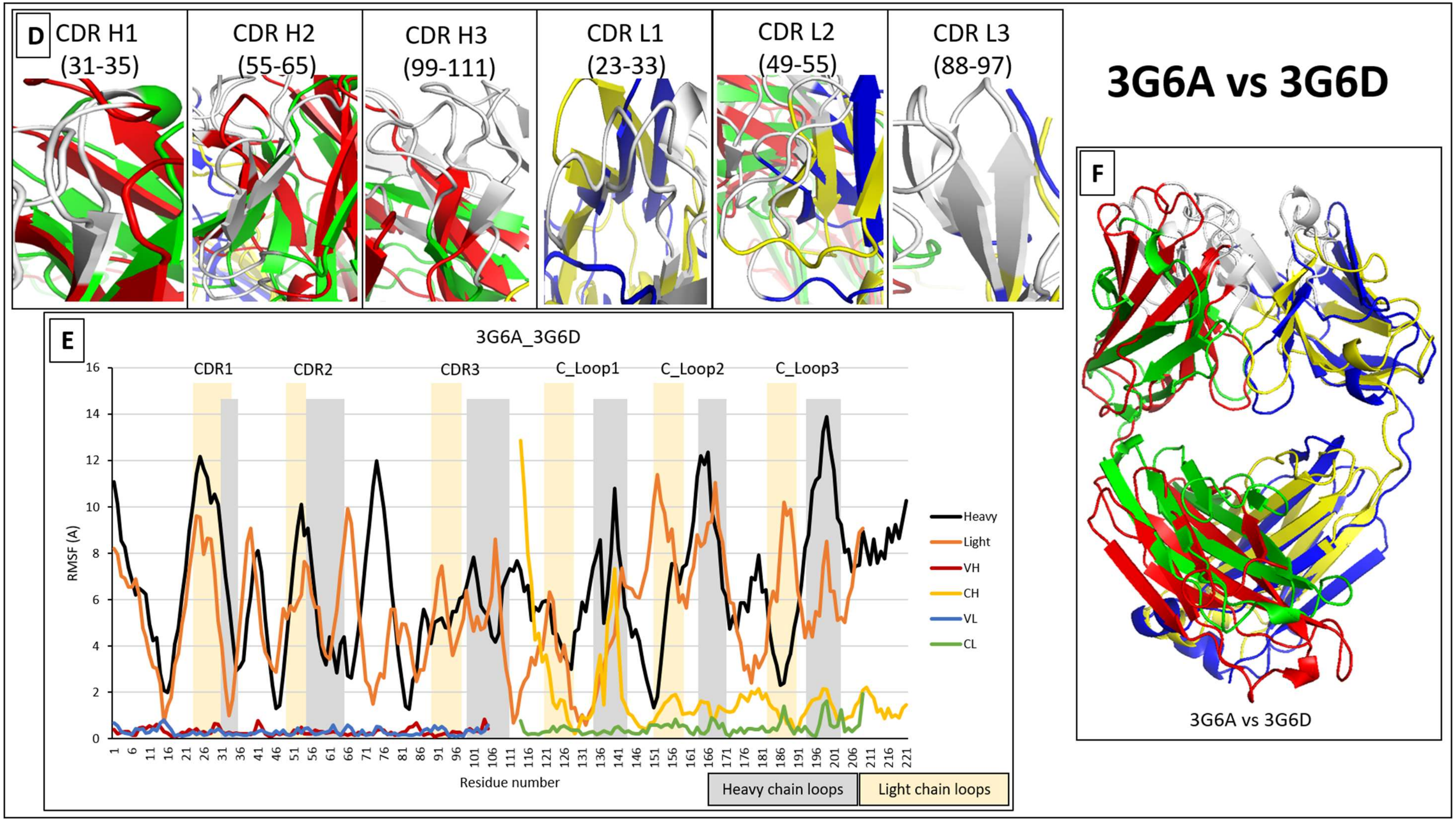

D
CDR H1 (31-35)
CDR H2 (55-65)
CDR H3 (99-111)
CDR L1 (23-33)
CDR L2 (49-55)
CDR L3 (88-97)
3G6A vs 3G6D
E
3G6A_3G6D
CDR1
CDR2
CDR3
C_Loop1
C_Loop2
C_Loop3
RMSF (A)
Residue number
Heavy
Light
VH
CH
VL
CL
Heavy chain loops
Light chain loops
F
3G6A vs 3G6D

S.2.4 (2FJF vs 2FJG), human:

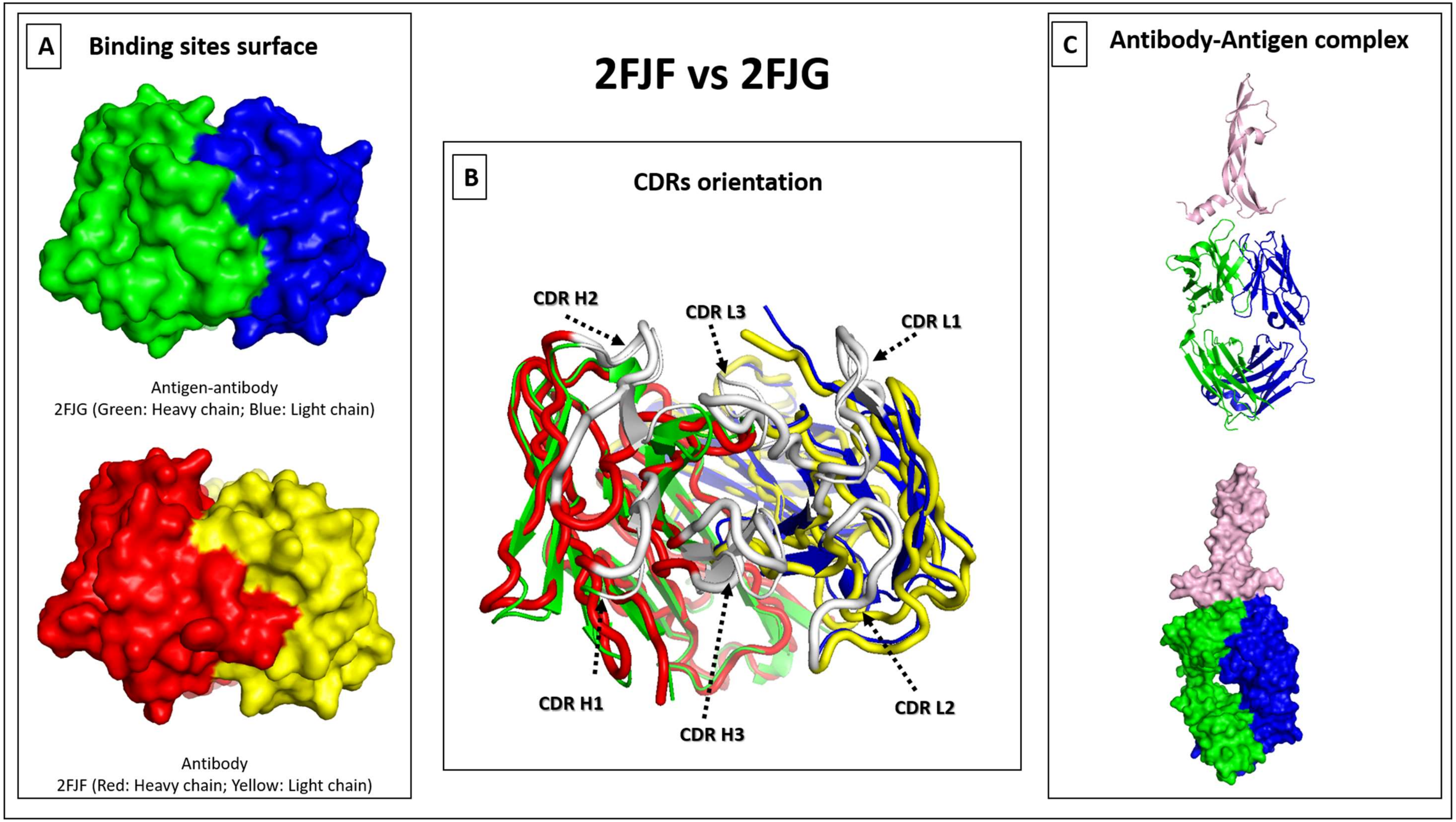

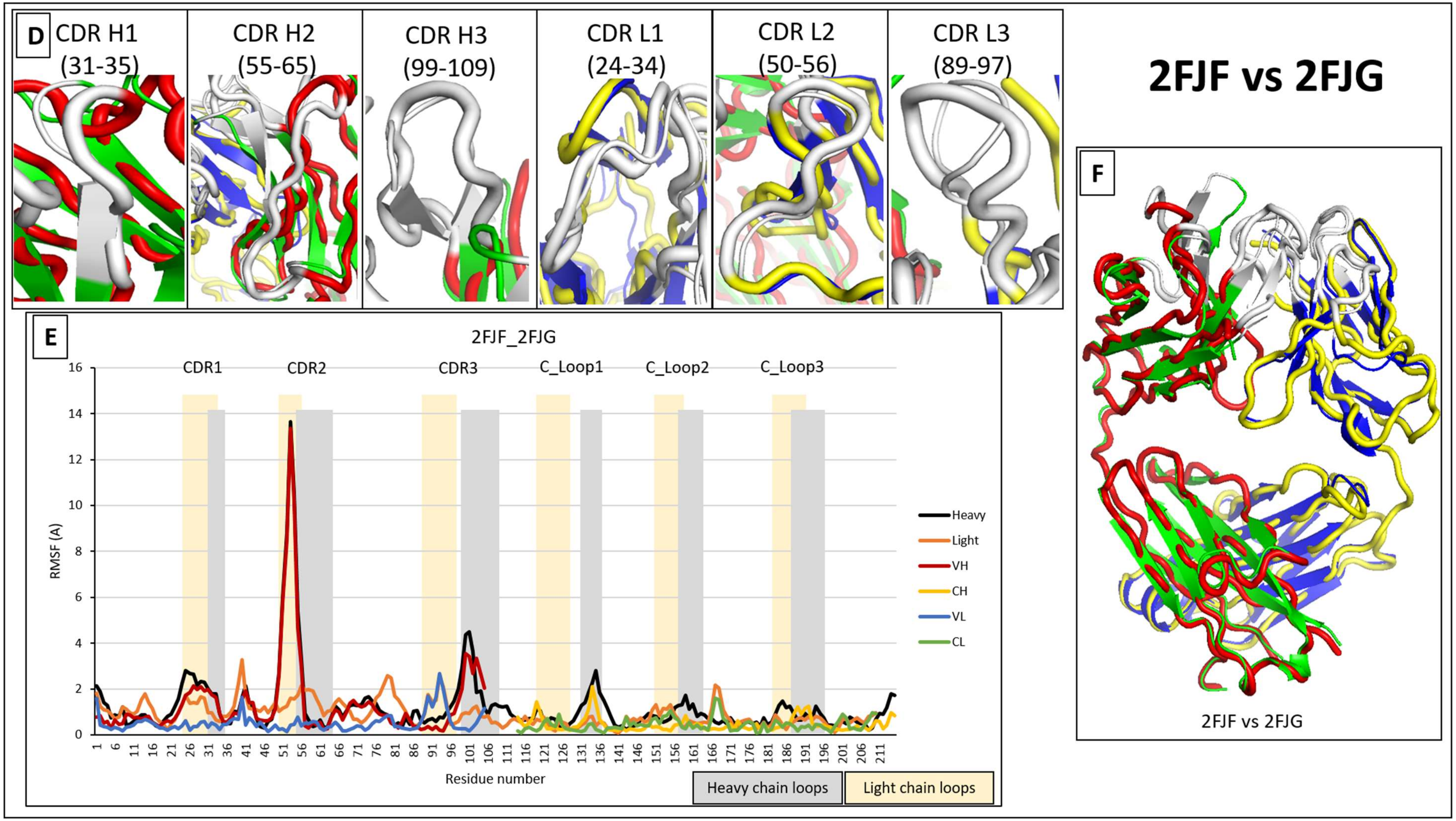
D
CDR H1 (31-35)
CDR H2 (55-65)
CDR H3 (99-109)
CDR L1 (24-34)
CDR L2 (50-56)
CDR L3 (89-97)
2FJF vs 2FJG
E
2FJF_2FJG
CDR1
CDR2
CDR3
C_Loop1
C_Loop2
C_Loop3
RMSF (A)
Residue number
Heavy
Light
VH
CH
VL
CL
Heavy chain loops
Light chain loops
F
2FJF vs 2FJG

S.2.5 (3HMX vs 3HMW), human:

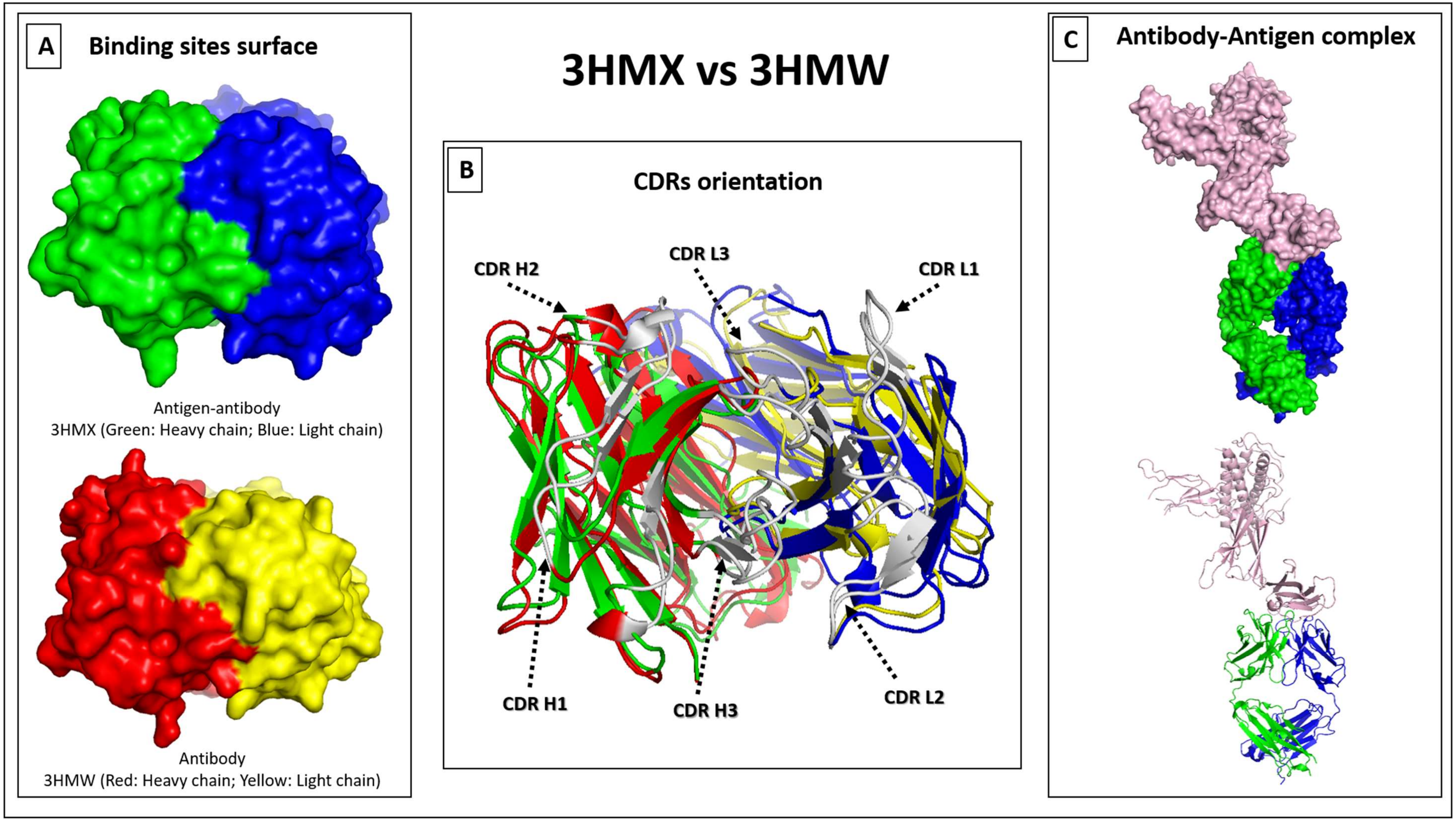

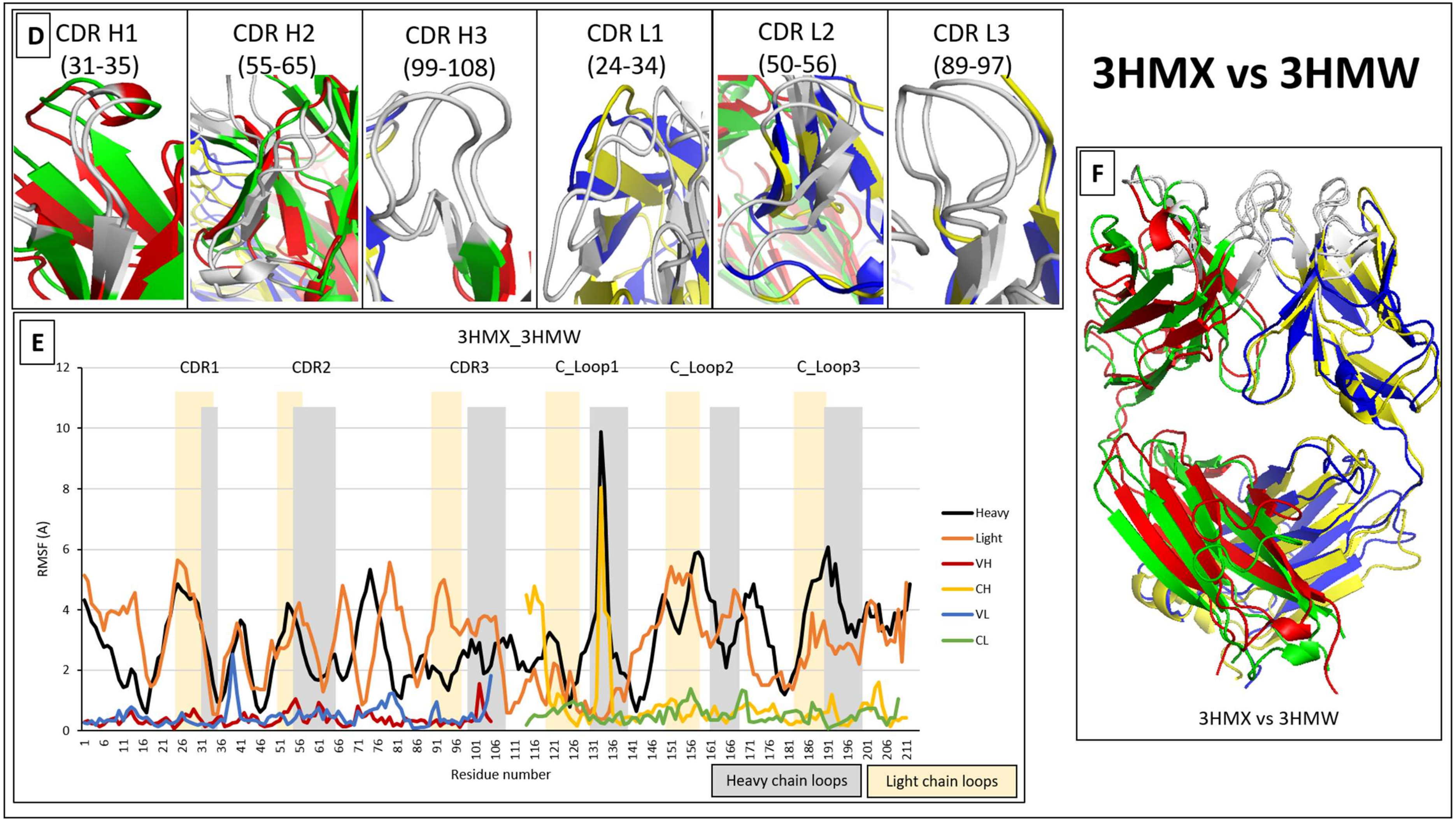
D
CDR H1 (31-35)
CDR H2 (55-65)
CDR H3 (99-108)
CDR L1 (24-34)
CDR L2 (50-56)
CDR L3 (89-97)
3HMX vs 3HMW
E
3HMX_3HMW
CDR1
CDR2
CDR3
C_Loop1
C_Loop2
C_Loop3
RMSF (A)
Residue number
Heavy
Light
VH
CH
VL
CL
Heavy chain loops
Light chain loops
F
3HMX vs 3HMW

S.2.6 (3EOA_3EO9), human:

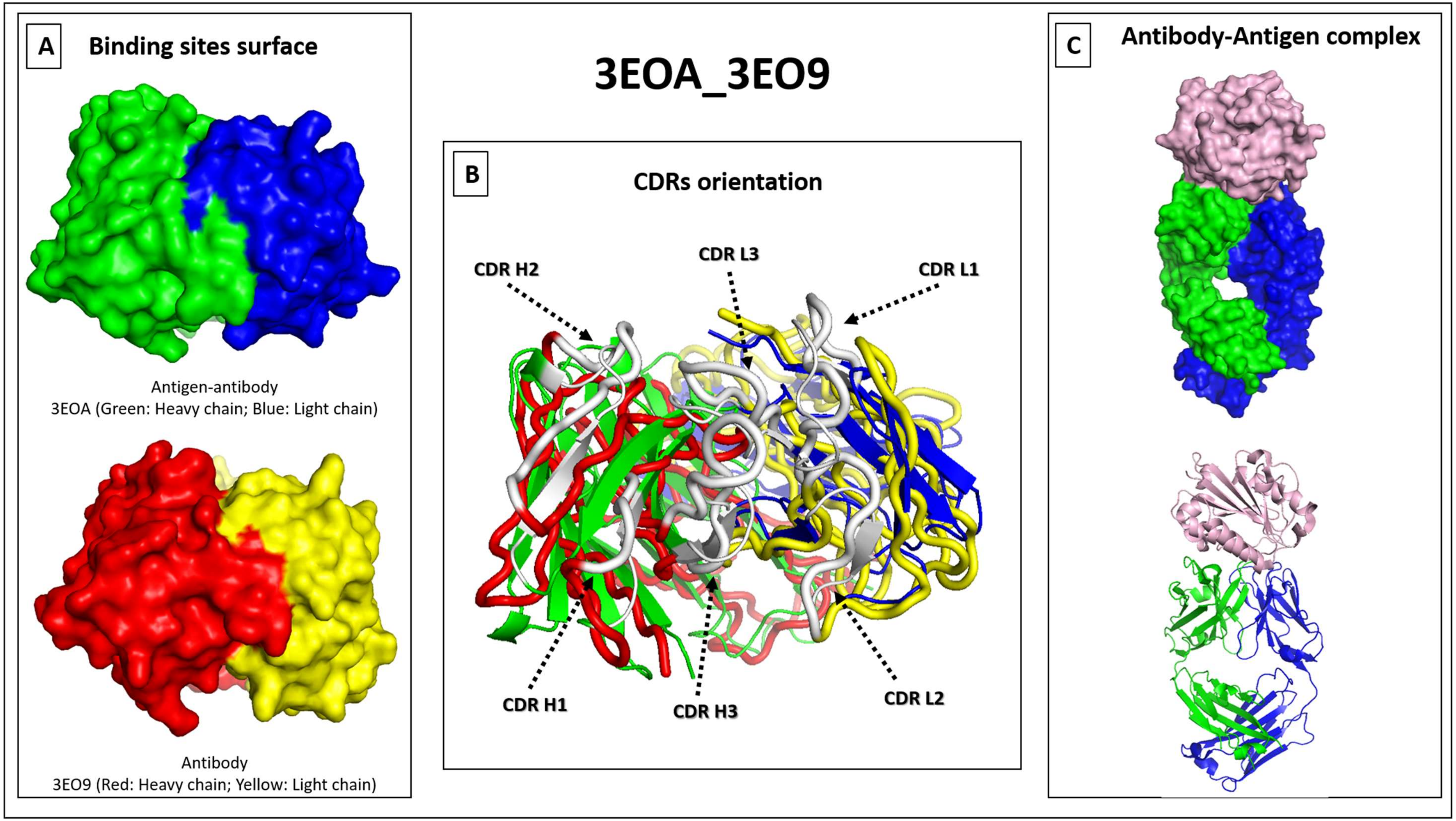

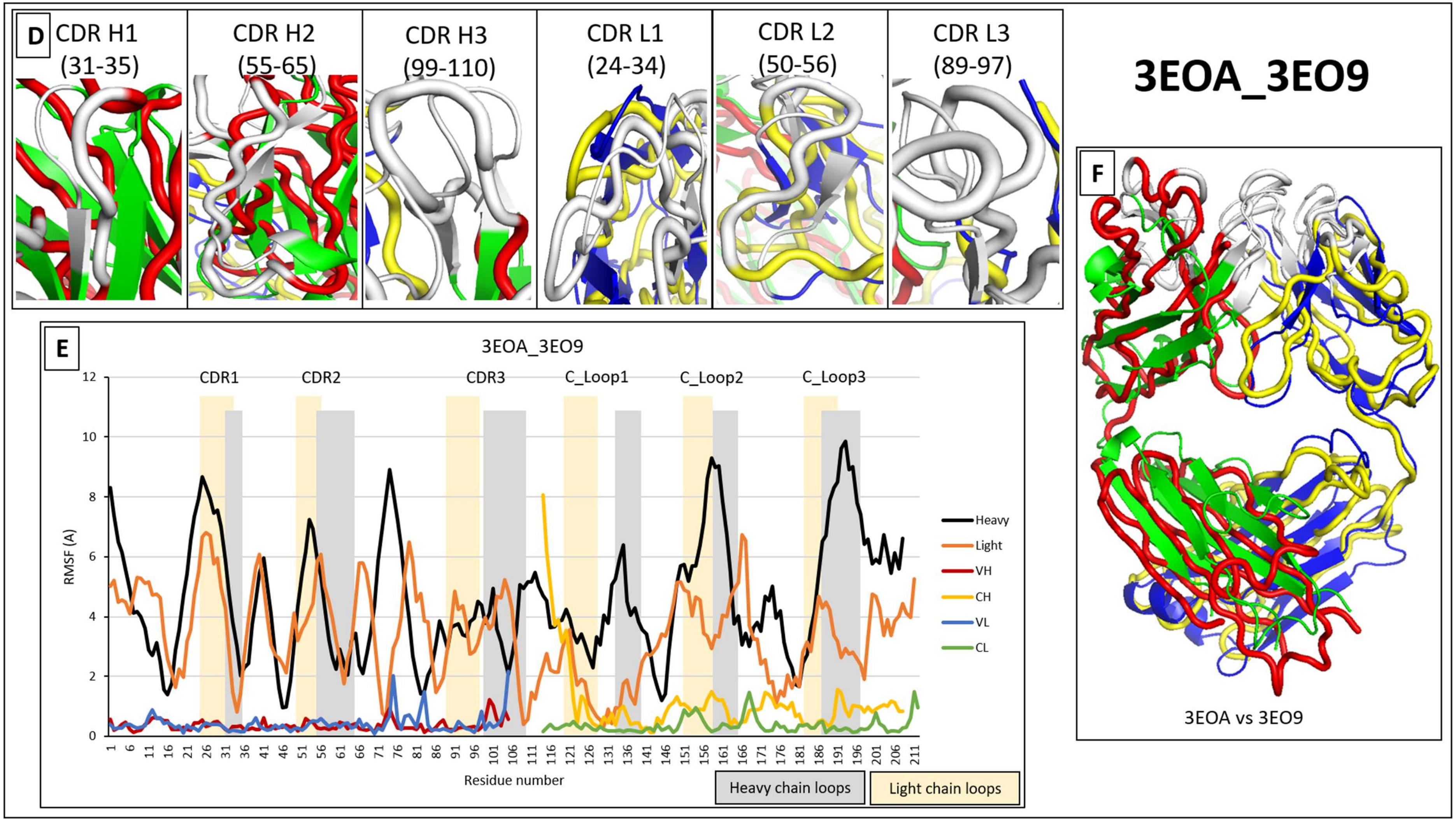
D
CDR H1 (31-35)
CDR H2 (55-65)
CDR H3 (99-110)
CDR L1 (24-34)
CDR L2 (50-56)
CDR L3 (89-97)
3EOA_3EO9
E
3EOA_3EO9
CDR1
CDR2
CDR3
C_Loop1
C_Loop2
C_Loop3
RMSF (A)
Residue number
Heavy
Light
VH
CH
VL
CL
Heavy chain loops
Light chain loops
F
3EOA vs 3EO9

S.2.7 (3EOB vs 3EO9), human:

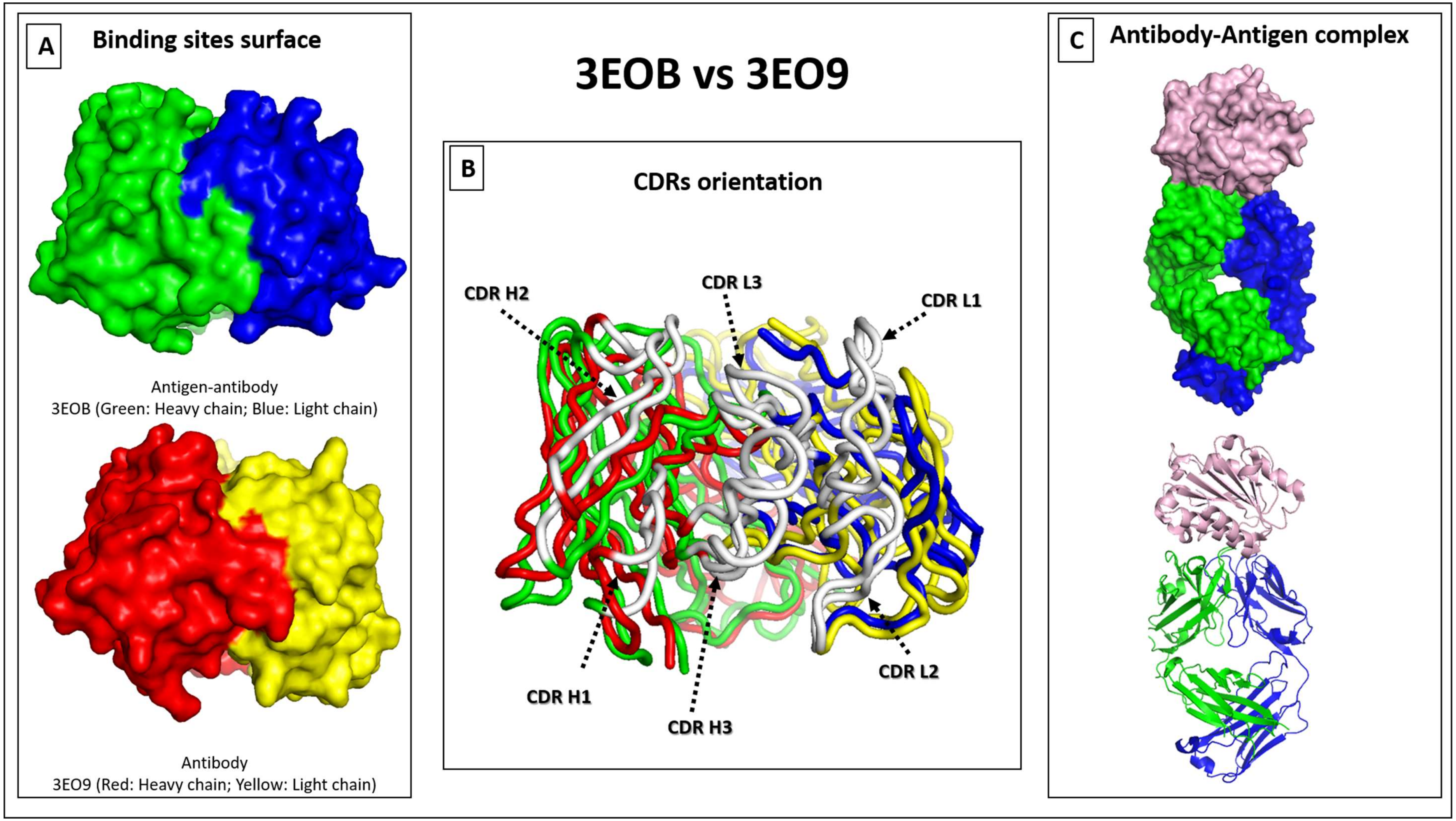

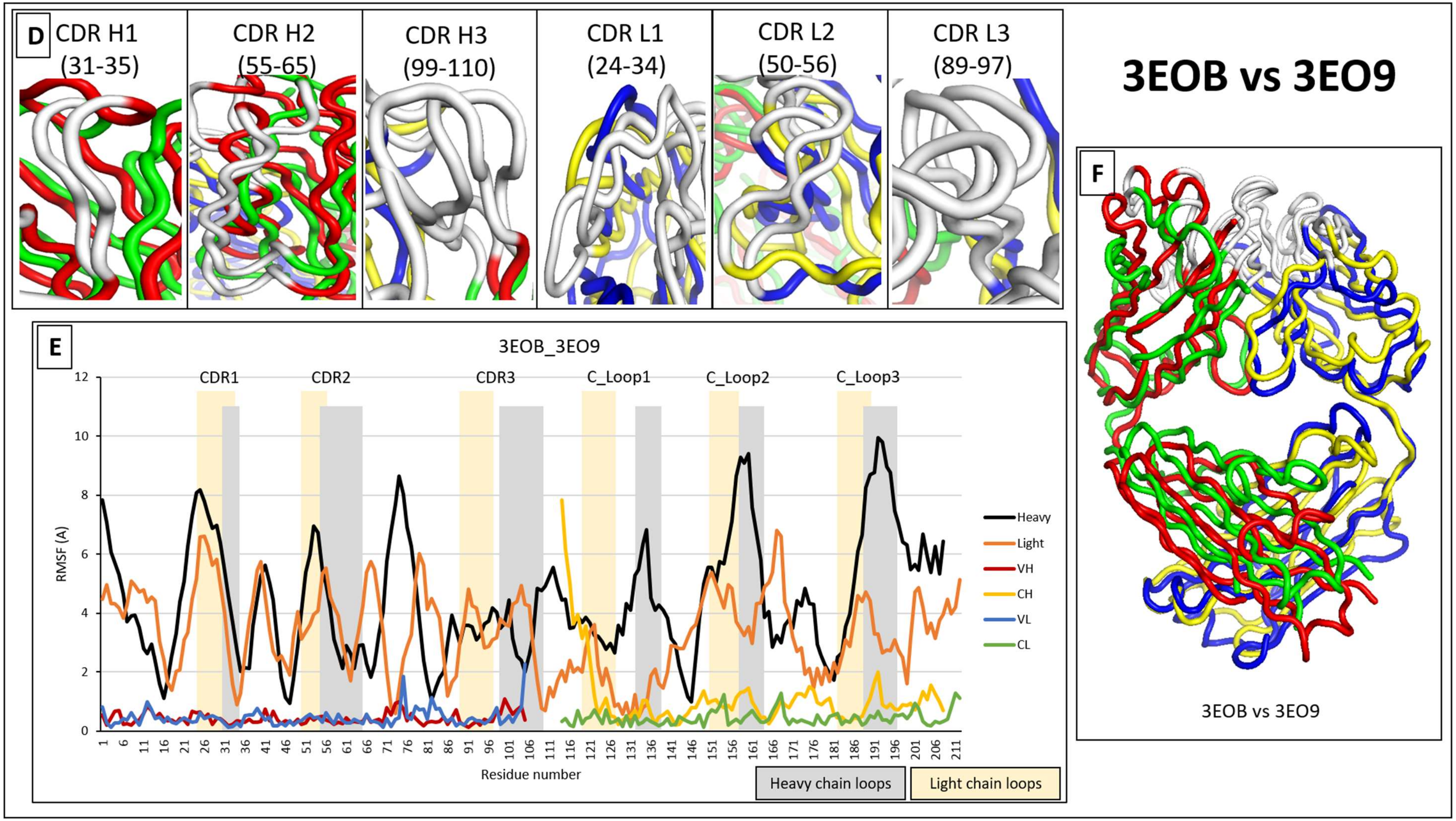
D
CDR H1 (31-35)
CDR H2 (55-65)
CDR H3 (99-110)
CDR L1 (24-34)
CDR L2 (50-56)
CDR L3 (89-97)
3EOB vs 3EO9
E
3EOB_3EO9
CDR1
CDR2
CDR3
C_Loop1
C_Loop2
C_Loop3
RMSF (A)
Residue number
Heavy
Light
VH
CH
VL
CL
Heavy chain loops
Light chain loops
F
3EOB vs 3EO9

S.2.8 (5BVP vs 5BVJ), human:

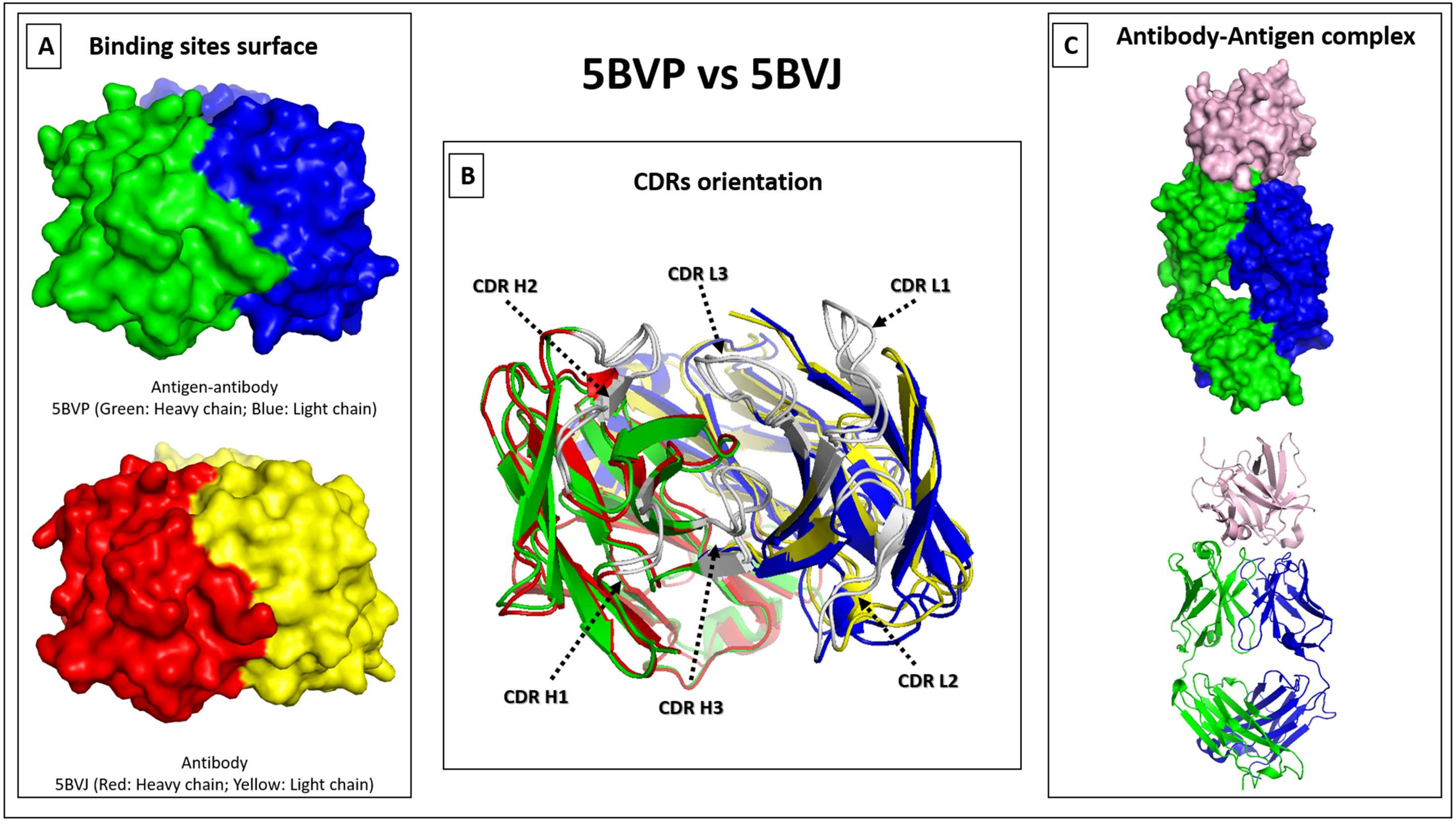

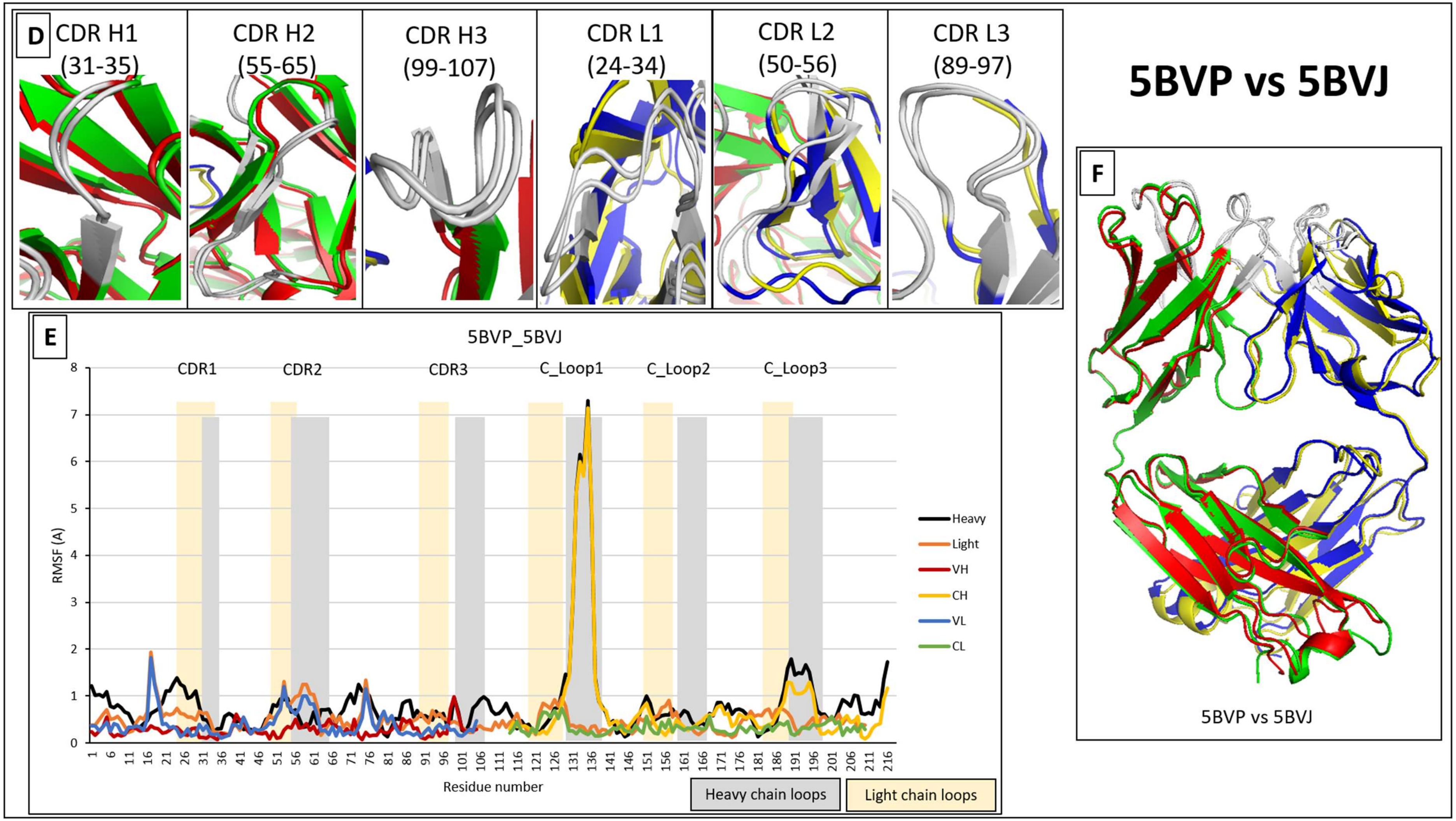
D
CDR H1 (31-35)
CDR H2 (55-65)
CDR H3 (99-107)
CDR L1 (24-34)
CDR L2 (50-56)
CDR L3 (89-97)
5BVP vs 5BVJ
E
5BVP_5BVJ
CDR1
CDR2
CDR3
C_Loop1
C_Loop2
C_Loop3
RMSF (A)
Residue number
Heavy
Light
VH
CH
VL
CL
Heavy chain loops
Light chain loops
F
5BVP vs 5BVJ

Supplementary S3: Sequences Alignment

The antibodies' sequences were acquired from the Protein Data Bank (PDB), and were analysed using BioEdit Sequence Alignment Editor, version 7.2.5*.A total of 11 human antibodies against protein antigens (4 mouse antibodies against protein antigens). Each sequence was split to heavy and light (lambda or kappa) chains, and then aligned. ClustalW Multiple alignment was used to align sequences of the same formats. Conserved positions of the aligned sequences to the first sequence were viewed by plotting identities a dot.

* Hall T. BioEdit: a user-friendly biological sequence alignment editor and analysis program for Windows 95/98/NT. Nucl Acids Symp Ser.1999;95–98.

Table S3.1 Protein_Human_Heavy chain_11 sequences

All constant domains (CH1) are of human IgG1 type.

```
                                   10        20        30        40        50        60        70        80        90       100
                          ....|....|....|....|....|....|....|....|....|....|....|....|....|....|....|....|....|....|....|....|
2FJF:B|PDBID|CHAIN|SEQUENCE EVQLVESGGGLVQPGGSLRLSCAASGFTISDYWIHWVRQAPGKGLEWVAGITPAGGYTYYADSVKGRFTISADTSKNTAYLQMNSLRAEDTAVYYCARFV
2FJG:B|PDBID|CHAIN|SEQUENCE ....................................................................................................
3EO9:H|PDBID|CHAIN|SEQUENCE ..........................YSFTGH.MN.............GM.H.SDSE.R.NQKF.D.....V.K....L...................GI
3EOA:B|PDBID|CHAIN|SEQUENCE ..........................YSFTGH.MN.............GM.H.SDSE.R.NQKF.D.....V.K....L...................GI
3EOB:B|PDBID|CHAIN|SEQUENCE ..........................YSFTGH.MN.............GM.H.SDSE.R.NQKF.D.....V.K....L...................GI
3G6A:H|PDBID|CHAIN|SEQUENCE Q...........................FNS...N.............S..AYDSSN.L............R.N....L...................GL
3G6D:H|PDBID|CHAIN|SEQUENCE Q...........................FNS...N.............S..AYDSSN.L............R.N....L...................GL
3HMW:H|PDBID|CHAIN|SEQUENCE .....Q..AEVKK..E..KI..KG..YSFTT..LG....M.....D.IGIMS.VDSDIR.SP.FQ.QV.M.V.K.IT.....W...K.S...M.....RR
3HMX:H|PDBID|CHAIN|SEQUENCE .....Q..AEVKK..E..KI..KG..YSFTT..LG....M.....D.IGIMS.VDSDIR.SP.FQ.QV.M.V.K.IT.....W...K.S...M.....RR
5BVJ:B|PDBID|CHAIN|SEQUENCE ..........V....R............F.V.GMN..............I.WYD.DNQ.............R.N....L.....G.............DL
5BVP:H|PDBID|CHAIN|SEQUENCE ..........V....R............F.V.GMN..............I.WYD.DNQ.............R.N....L.....G.............DL

                                  110       120       130       140       150       160       170       180       190       200
                          ....|....|....|....|....|....|....|....|....|....|....|....|....|....|....|....|....|....|....|....|
2FJF:B|PDBID|CHAIN|SEQUENCE FFLPYAM--DYWGQGTLVTVSSASTKGPSVFPLAPSSKSTSGGTAALGCLVKDYFPEPVTVSWNSGALTSGVHTFPAVLQSSGLYSLSSVVTVPSSSLGT
2FJG:B|PDBID|CHAIN|SEQUENCE .......--...........................................................................................
3EO9:H|PDBID|CHAIN|SEQUENCE Y.YGTTY-F...........................................................................................
3EOA:B|PDBID|CHAIN|SEQUENCE Y.YGTTY-F...........................................................................................
3EOB:B|PDBID|CHAIN|SEQUENCE Y.YGTTY-F...........................................................................................
3G6A:H|PDBID|CHAIN|SEQUENCE GAFHWD.QP...........................................................................................
3G6D:H|PDBID|CHAIN|SEQUENCE GAFHWD.QP...........................................................................................
3HMW:H|PDBID|CHAIN|SEQUENCE PGQG.---F.F...........S.............................................................................
3HMX:H|PDBID|CHAIN|SEQUENCE PGQG.---F.F...........S.............................................................................
5BVJ:B|PDBID|CHAIN|SEQUENCE RTG.F----...........................................................................................
5BVP:H|PDBID|CHAIN|SEQUENCE RTG.F----...........................................................................................

                                  210       220
                          ....|....|....|....|....
2FJF:B|PDBID|CHAIN|SEQUENCE QTYICNVNHKPSNTKVDKKVEPKSCDKTH
2FJG:B|PDBID|CHAIN|SEQUENCE .............................
3EO9:H|PDBID|CHAIN|SEQUENCE ....................---------
3EOA:B|PDBID|CHAIN|SEQUENCE .....................--------
3EOB:B|PDBID|CHAIN|SEQUENCE .....................--------
3G6A:H|PDBID|CHAIN|SEQUENCE .............................
3G6D:H|PDBID|CHAIN|SEQUENCE .............................
3HMW:H|PDBID|CHAIN|SEQUENCE ..................R..........
3HMX:H|PDBID|CHAIN|SEQUENCE ..................R..........
5BVJ:B|PDBID|CHAIN|SEQUENCE ..................R..........
5BVP:H|PDBID|CHAIN|SEQUENCE ..................R..........
```

Table S3.2 Protein_Human_Light chain_Kappa_9 sequences

All constant domains (Ck) are of human kappa type

```
                                    10        20        30        40        50        60        70        80        90       100
                           ....|....|....|....|....|....|....|....|....|....|....|....|....|....|....|....|....|....|....|....|
2FJF:A|PDBID|CHAIN|SEQUENCE DIQMTQSPSSLSASVGDRVTITCRASQDVSTAVAWYQQKPGKAPKLLIYSASFLYSGVPSRFSGSGSGTDFTLTISSLQPEDFATYYCQQSYTTPPTFGQ
2FJG:A|PDBID|CHAIN|SEQUENCE ....................................................................................................
3EO9:L|PDBID|CHAIN|SEQUENCE ..........................KTI.KYL.................G.T.Q...................................HNEY.L....
3EOA:A|PDBID|CHAIN|SEQUENCE ..........................KTI.KYL.................G.T.Q...................................HNEY.L....
3EOB:A|PDBID|CHAIN|SEQUENCE ..........................KTI.KYL.................G.T.Q...................................HNEY.L....
3HMW:L|PDBID|CHAIN|SEQUENCE ...........................GI.SWL.......E....S...A..S.Q...................................YNIY.Y....
3HMX:L|PDBID|CHAIN|SEQUENCE ...........................GI.SWL.......E....S...A..S.Q...................................YNIY.Y....
5BVJ:E|PDBID|CHAIN|SEQUENCE E.VL....DFQ.VTPKEK.........SIGSSLH......DQS.....KY..QSF....................N..EA..A.A...H..SSL.F...P
5BVP:L|PDBID|CHAIN|SEQUENCE E.VL....DFQ.VTPKEK.........SIGSSLH......DQS.....KY..QSF....................N..EA..A.A...H..SSL.F...P

                                   110       120       130       140       150       160       170       180       190       200
                           ....|....|....|....|....|....|....|....|....|....|....|....|....|....|....|....|....|....|....|....|
2FJF:A|PDBID|CHAIN|SEQUENCE GTKVEIKRTVAAPSVFIFPPSDEQLKSGTASVVCLLNNFYPREAKVQWKVDNALQSGNSQESVTEQDSKDSTYSLSSTLTLSKADYEKHKVYACEVTHQG
2FJG:A|PDBID|CHAIN|SEQUENCE ....................................................................................................
3EO9:L|PDBID|CHAIN|SEQUENCE ....................................................................................................
3EOA:A|PDBID|CHAIN|SEQUENCE ....................................................................................................
3EOB:A|PDBID|CHAIN|SEQUENCE ....................................................................................................
3HMW:L|PDBID|CHAIN|SEQUENCE ..L.................................................................................................
3HMX:L|PDBID|CHAIN|SEQUENCE ..L.................................................................................................
5BVJ:E|PDBID|CHAIN|SEQUENCE ....D...............................................................................................
5BVP:L|PDBID|CHAIN|SEQUENCE ....D...............................................................................................

                                   210
                           ....|....|....
2FJF:A|PDBID|CHAIN|SEQUENCE LSSPVTKSFNRGEC
2FJG:A|PDBID|CHAIN|SEQUENCE ..............
3EO9:L|PDBID|CHAIN|SEQUENCE ..............
3EOA:A|PDBID|CHAIN|SEQUENCE ..............
3EOB:A|PDBID|CHAIN|SEQUENCE ..............
3HMW:L|PDBID|CHAIN|SEQUENCE ..............
3HMX:L|PDBID|CHAIN|SEQUENCE ..............
5BVJ:E|PDBID|CHAIN|SEQUENCE ..............
5BVP:L|PDBID|CHAIN|SEQUENCE ..............
```

Table S3.3 Protein_Human_Light_Lambda_2 sequences

All constant domains (Cλ) are of human lambda type

```
                                    10        20        30        40        50        60        70        80        90       100
                            ....|....|....|....|....|....|....|....|....|....|....|....|....|....|....|....|....|....|....|....|
3G6A:L|PDBID|CHAIN|SEQUENCE SYELTQPPSVSVAPGQTARISCSGDNIGGTFVSWYQQKPGQAPVLVIYDDNDRPSGIPERFSGSNSGNTATLTISGTQAEDEADYYCGTWDMVTNNVFGG
3G6D:L|PDBID|CHAIN|SEQUENCE ....................................................................................................

                                   110       120       130       140       150       160       170       180       190       200
                            ....|....|....|....|....|....|....|....|....|....|....|....|....|....|....|....|....|....|....|....|
3G6A:L|PDBID|CHAIN|SEQUENCE GTKLTVLGQPKAAPSVTLFPPSSEELQANKATLVCLISDFYPGAVTVAWKADSSPVKAGVETTTPSKQSNNKYAASSYLSLTPEQWKSHRSYSCQVTHEG
3G6D:L|PDBID|CHAIN|SEQUENCE ....................................................................................................

                                   210
                            ....|....|...
3G6A:L|PDBID|CHAIN|SEQUENCE STVEKTVAPTECS
3G6D:L|PDBID|CHAIN|SEQUENCE .............
```

Table S3.4 Protein_Mouse_Heavy chain_4 sequences

The 4 sequences can be classified as 2 IgG1 (1DQJ and 1DQQ), and 2 IgG2a (1MLC and 1MLB).

```
                                     10        20        30        40        50        60        70        80        90       100
                              ....|....|....|....|....|....|....|....|....|....|....|....|....|....|....|....|....|....|....|....|
1DQJ:B|PDBID|CHAIN|SEQUENCE   EVQLQESGPSLVKPSQTLSLTCSVTGDSVTSDYWSWIRKFPGNKLEYMGYIS-YSGSTYYHPSLKSRISITRDTSKNQYYLQLNSVTTEDTATYYCASWG
1DQQ:B|PDBID|CHAIN|SEQUENCE   ....................................................-...............................................
1MLB:B|PDBID|CHAIN|SEQUENCE   Q.......AEVM..GASVKIS.KA..YTFSTYWIE.VKQR..HG..WI.E.LPG......NEKF.GKATF.A...S.TA.M..S.L.S..S.V....RGD
1MLC:B|PDBID|CHAIN|SEQUENCE   Q.......AEVM..GASVKIS.KA..YTFSTYWIE.VKQR..HG..WI.E.LPG......NEKF.GKATF.A...S.TA.M..S.L.S..S.V....RGD

                                    110       120       130       140       150       160       170       180       190       200
                              ....|....|....|....|....|....|....|....|....|....|....|....|....|....|....|....|....|....|....|....|
1DQJ:B|PDBID|CHAIN|SEQUENCE   G--DVWGAGTTVTVSSAKTTAPSVYPLAPVCGDTTGSSVTLGCLVKGYFPEPVTLTWNSGSLSSGVHTFPAVLQSDLYTLSSSVTVTSSTWPSQSITCNV
1DQQ:B|PDBID|CHAIN|SEQUENCE   .--.................................................................................................
1MLB:B|PDBID|CHAIN|SEQUENCE   .NYGY..Q...L.....S..P...F....GSAAQ.N.M................V...............................P..PR..ETV....
1MLC:B|PDBID|CHAIN|SEQUENCE   .NYGY..Q...L.....S..P...F....GSAAQ.N.M................V...............................P..PR..ETV....

                                    210
                              ....|....|....|...
1DQJ:B|PDBID|CHAIN|SEQUENCE   AHPASSTKVDKKI-----
1DQQ:B|PDBID|CHAIN|SEQUENCE   .............-----
1MLB:B|PDBID|CHAIN|SEQUENCE   .............VPRDC
1MLC:B|PDBID|CHAIN|SEQUENCE   .............VPRDC
```

Table S3.5 Protein_Mouse_Light_Kappa_4 sequences

All constant domains (Ck) are of mouse kappa type.

```
                                  10        20        30        40        50        60        70        80        90       100
                          ....|....|....|....|....|....|....|....|....|....|....|....|....|....|....|....|....|....|....|....|
1DQJ:A|PDBID|CHAIN|SEQUENCE  DIVLTQSPATLSVTPGDSVSLSCRASQSISNNLHWYQQKSHESPRLLIKYASQSISGIPSRFSGSGSGTDFTLSINSVETEDFGMYFCQQSNSWPYTFGG
1DQQ:A|PDBID|CHAIN|SEQUENCE  ....................................................................................................
1MLB:A|PDBID|CHAIN|SEQUENCE  ..E...............................................V...S........................................R....
1MLC:A|PDBID|CHAIN|SEQUENCE  ..E...............................................V...S........................................R....

                                 110       120       130       140       150       160       170       180       190       200
                          ....|....|....|....|....|....|....|....|....|....|....|....|....|....|....|....|....|....|....|....|
1DQJ:A|PDBID|CHAIN|SEQUENCE  GTKLEIKRADAAPTVSIFPPSSEQLTSGGASVVCFLNNFYPKDINVKWKIDGSERQNGVLNSWTDQDSKDSTYSMSSTLTLTKDEYERHNSYTCEATHKT
1DQQ:A|PDBID|CHAIN|SEQUENCE  ....................................................................................................
1MLB:A|PDBID|CHAIN|SEQUENCE  ....................................................................................................
1MLC:A|PDBID|CHAIN|SEQUENCE  ....................................................................................................

                                 210
                          ....|....|....
1DQJ:A|PDBID|CHAIN|SEQUENCE  STSPIVKSFNRNEC
1DQQ:A|PDBID|CHAIN|SEQUENCE  ..............
1MLB:A|PDBID|CHAIN|SEQUENCE  ..............
1MLC:A|PDBID|CHAIN|SEQUENCE  ..............
```

Supplementary S4: Angles and distances

Angles and domains packing were measured as described in the Methods section. Distances were measured in angstrom (Å).

PDB number	PDB ID	Crystal form	Linker to linker distance	c-c (light)	c-c (heavy)	Light angle	Heavy angle	Magnitude of Average Angle Change
1	1MLC	Antigen-antibody	39.5	41.3	38.1	104.1	93.4	3.5
2	1MLB	Antibody	40.1	42	36.8	107.4	89.7	
3	1DQJ	Antigen-antibody	40.1	38.2	39.1	94.4	99.2	3.5
4	1DQQ	Antibody	39.9	39	38.1	97.8	95.6	
5	3G6D	Antigen-antibody	39.8	45.2	26.9	114.2	60.3	37.2
6	3G6A	Antibody	38.1	36.1	41.2	84.9	105.4	
7	2FJG	Antigen-antibody	40.8	42.7	33.1	110.4	76.8	3.2

8	2FJF	Antibody	40.6	42.7	34.2	108	80.8	
9	3HMX	Antigen-antibody	40.9	44.8	30.5	116.1	70.8	16.55
10	3HMW	Antibody	40.1	40.3	35.9	100	87.8	
11	3EOA	Antigen-antibody	40.8	44	28.7	113.4	65.7	24.9
12	3EOB	Antigen-antibody	40.7	44	28.9	113.4	65.9	24.8
13	3EO9	Antibody	39.2	39	39.5	96.8	98.9	
14	5BVP	Antigen-antibody	40.4	37.6	38.3	93.2	94.8	2.4
15	5BVJ	Antibody	40.5	38.2	37.1	94.4	91.2	
